# *Akkermansia muciniphila* protects the intestine from irradiation-induced injury by secretion of propionic acid

**DOI:** 10.1080/19490976.2023.2293312

**Published:** 2023-12-12

**Authors:** Kai-Yue He, Xin-Yuan Lei, Dan-Hui Wu, Lei Zhang, Jun-Qi Li, Qiu-Tong Li, Wei-Tao Yin, Zi-Long Zhao, Huai Liu, Xiong-Yan Xiang, Ling-Jun Zhu, Cui-Yun Cui, Ke-Ke Wang, Jin-Hua Wang, Lin Lv, Qian-Hui Sun, Guo-Long Liu, Zhi-Xiang Xu, Yong-Ping Jian

**Affiliations:** a School of Life Sciences, Henan University, Kaifeng, Henan, China; b Department of Blood Transfusion, Henan Provincial People’s Hospital, Zhengzhou, Henan, China; c Jiangsu Cancer Hospital, Jiangsu Institute of Cancer Research, and The Affiliated Cancer Hospital of Nanjing Medical University, Nanjing, Jiangsu, China; d Department of Medical Oncology, Guangzhou First People’s Hospital, School of Medicine, South China University of Technology, Guangzhou, Guangdong, China

**Keywords:** Irradiation-induced enteritis, akkermansia muciniphila, propionic acid, intestinal epithelial barrier, metformin, histone acetylation

## Abstract

Intestinal dysbiosis frequently occurs in abdominal radiotherapy and contributes to irradiation (IR)-induced intestinal damage and inflammation. *Akkermansia muciniphila* (*A. muciniphila*) is a recently characterized probiotic, which is critical for maintaining the dynamics of the intestinal mucus layer and preserving intestinal microbiota homeostasis. However, the role of *A. muciniphila* in the alleviation of radiation enteritis remains unknown. In this study, we reported that the abundance of *A. muciniphila* was markedly reduced in the intestines of mice exposed to abdominal IR and in the feces of patients who received abdominal radiotherapy. Abundance of *A. muciniphila* in feces of radiotherapy patients was negatively correlated with the duration of diarrhea in patients. Administration of *A. muciniphila* substantially mitigated IR-induced intestinal damage and prevented mouse death. Analyzing the metabolic products of *A. muciniphila* revealed that propionic acid, a short-chain fatty acid secreted by the microbe, mediated the radioprotective effect. We further demonstrated that propionic acid bound to G-protein coupled receptor 43 (GRP43) on the surface of intestinal epithelia and increased histone acetylation and hence enhanced the expression of tight junction proteins occludin and ZO-1 and elevated the level of mucins, leading to enhanced integrity of intestinal epithelial barrier and reduced radiation-induced intestinal damage. Metformin, a first-line agent for the treatment of type II diabetes, promoted intestinal epithelial barrier integrity and reduced radiation intestinal damage through increasing the abundance of *A. muciniphila*. Together, our results demonstrated that *A. muciniphila* plays a critical role in the reduction of abdominal IR-induced intestinal damage. Application of probiotics or their regulators, such as metformin, could be an effective treatment for the protection of radiation exposure-damaged intestine.

## Introduction

Radiotherapy is indispensable for a comprehensive treatment of cancer, and enteritis is the most common complication of abdominal radiotherapy^1^. Acute enteritis occurs in 50% to 70% of the patients, who received abdominal or pelvic radiotherapy. Abdominal irradiation (IR) destroys the intestinal epithelial cells, leading to elevated permeabilization of the intestinal epithelial barrier, intestinal infection, and multiple-organ dysfunction syndrome^2^.

Gut microorganisms, mainly composed of bacteria, fungi, viruses, and protists, co-exist and co-evolve with the host^3^. Gut microbiota, which is normally between 10^10^ and 10^11^ microbial cells per wet-weight gram of feces in the intestine, weights less than 500 g in an adult^4,5^. The number of intestinal flora is close to a ratio of 1:1 with that of human cells^6^. Based on the spatial distribution, gut microbiota is divided into mucus layer flora and intestinal cavity flora. Both play an important role in nutrient exchange, immune system development, signal transduction, and defenses against pathogen invasion^7,8^. *Lactobacillus* dominates in intestinal cavity bacteria, whereas *Bacteroidia*, *Clostridia*, and *Akkermansia muciniphila* (*A. muciniphila*) mainly colonize in the mucus layer^9^.

By transplanting microbiota from mice with IR-induced intestine injury to germ-free and dextran sodium sulfate (DSS)-induced colitis mice, Gerassy-Vainberg et al. demonstrated that radiotherapy-induced dysbiosis contributes to the development of IR-induced intestinal damage and susceptibility of mice to intestinal inflammation^10^. Cui et al. also reported that fecal microbiota transplantation (FMT) reduces the gastrointestinal toxicity caused by radiotherapy and protects the intestinal epithelia from radiotherapy-induced damage^11^. Uribe et al. reported that *Lactobacillus rhamnosus GG* maintains intestinal homeostasis by increasing the expression of cyclooxygenase-2 (COX-2) and secretion of prostaglandin E2 in colonic myofibroblasts in a MyD88-dependent manner^4^. In addition, *Lactobacillus* was found to protect the intestinal epithelium from IR-induced injury by promoting the activation of toll like receptor 2 (TLR-2)/COX-2, release of lipoteichoic acid, activation of macrophage, and migration of mesenchymal stem cells^5,12^. Together, current studies suggest that IR leads to intestinal dysbiosis, which possibly contributes to IR-induced intestinal epithelial damage. Correction of the intestinal dysbiosis could be an effective strategy for the treatment of IR-induced intestinal injury^11^.

*A. muciniphila*, a member of the *Verrucomicrobia phylum*, is a gram-negative anaerobic bacterium. It preferentially colonizes in mucins-rich intestinal mucus layers, in which it specifically degrades mucins as carbon, nitrogen and energy sources for itself and for the host^13^. *A. muciniphila* also degrades oligosaccharides in the milk to generate propionic acid to promote self-colonization. *A. muciniphila-*produced short-chain fatty acids (SCFAs) stimulate the growth and metabolism of bacteria that colonize in the mucus layers and prevent pathogenic bacteria passing through the mucus layers to destroy intestinal epithelial cells (IECs)^14^. Thus, it plays a protective role in the maintenance of intestinal barrier integrity. However, it has not been explored whether *A. muciniphila* is altered in abdominal IR-induced intestinal damage and plays a role in the alleviation of the damage.

Metformin, a first line drug for the treatment of type II diabetes, was previously found to reduce the blood glucose of diabetic patients by maintaining a balance of gut commensal microorganisms^15,16^. We recently reported that metformin improved the composition of gut microbiota in patients receiving abdominal IR^17^. Abundance of *A. muciniphila* was markedly increased in radiotherapy patients and in abdominal IR mice treated with metformin^17^, indicating the potential application of metformin in abdominal IR subjects.

Although *A. muciniphila*, propionic acid, and metformin have individually been reported to decrease intestinal inflammation, it remains unknown whether these agents could alleviate radiation enteritis and if propionic acid plays a role in the mediation of the probiotic function of *A. muciniphila*. Additionally, the functional outcomes of metformin with the increase of *A. muciniphila* abundance and alleviation of radiation enteritis are undefined. In the current study, we identified that *A. muciniphila* was substantially decreased in the intestine of mice and in patients received abdominal IR. Administration of *A. muciniphila* alone was sufficient to lessen IR-induced intestinal injury in mice and prevented mice from IR enteritis-associated death. We presented evidence showing that propionic acid secreted by *A. muciniphila* mediated the protective effect. Metformin promoted the integrity of intestinal epithelial barrier and reduced radiation intestinal damage by elevating the abundance of *A. muciniphila* in intestine of abdominal IR mice. Together, our findings provide new insights for the prevention and treatment of IR-induced intestinal injury.

## Results

### Gut dysbiosis in patients with abdominal radiotherapy

**Figure 1.**
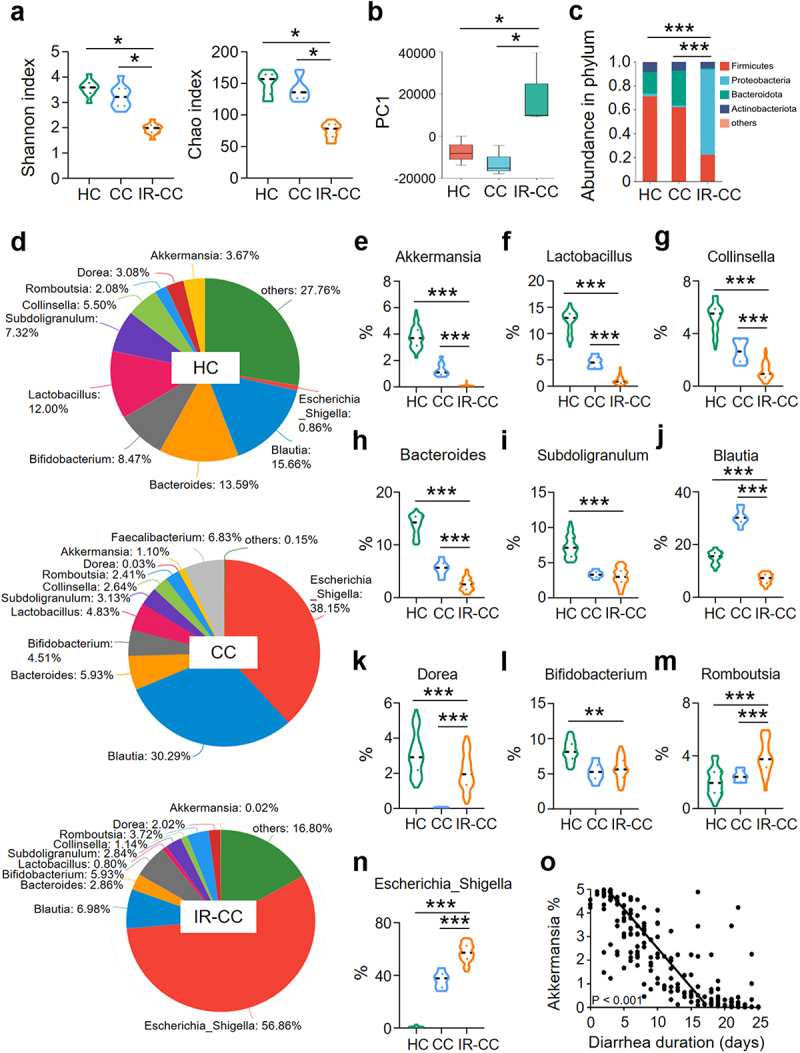
Abundance of *A. muciniphila* is reduced in stool from cervical cancer patients with radiotherapy.

To determine the impact of IR on intestinal flora, we collected fecal samples from cervical cancer patients with (IR-CC) or without (CC) abdominal radiotherapy and from age-matched healthy subjects (HC) for 16S rRNA sequencing. The diversity of the gut microbiota was markedly decreased in IR-CC based on the Shannon index analysis (Figure 1a). The abundance of intestinal flora was also reduced in IR-CC based on the Chao index analysis (Figure 1a). A relatively close distance between HC and CC was observed, suggesting that the composition of microbiota in the two groups bore a similar pattern, whereas the distance between IR-CC and CC was relatively farther, suggesting a less similarity in the composition of flora in the two groups (Figure 1b). Together, these results indicate that IR leads to the gut dysbiosis in cervical cancer patients.

Stool samples from cervical cancer patients with or without abdominal IR and from healthy subjects were collected. Total DNAs were isolated from the feces as described in Materials and Methods and subjected to 16S rRNA sequencing. (a) Shannon index and chao index of intestinal flora. (b) PCoA analysis. (c) Composition on phylum level. (d) Pie analysis of intestinal flora in healthy subjects, CC patients and IR-CC patients. (e-n) Percentage of *A. muciniphila* (e), *Lactobacillus* (f), *Collinsella* (g), *Bacteroides* (h), *Subdoligranulum* (i), *Blautia* (j), *Dorea* (k), *Bifidobacterium* (l), *Romboutsia* (m) and *Escherichia-Shigella* (n) in the intestinal flora. (o) Correlation between the abundance of *A. muciniphila* in feces of radiotherapy patients and the duration of diarrhea in patients. ** *P* < 0.01, *** *P* < 0.001. Data represent mean ± SEM, compared with HC or CC, *n* = 83 in HC, *n* = 78 in CC and *n* = 87 in IR-CC.

The composition of gut microbiota in IR-CC, CC, and HC was quite different (Figure 1c-d). Abundance of *Proteobacteria* was augmented, whereas that of *Firmicutes* and *Bacteroidota* were reduced in IR-CC, as compared with those in CC or HC (Figure 1c). At the genus level, the abundance of *Romboutsia* and *Escherichia-Shigella* was increased (Figure 1m-n), whereas the abundance of *A. muciniphila*, *Lactobacillus*, *Collinsella*, *Bacteroides*, and *Blautia* was decreased in IR-CC (Figure 1d-n). Among them, reduction of *A. muciniphila* was mostly substantial in IR-CC, as compared with that in CC or HC (Figure 1e). Similar results were obtained when we monitored the gut flora of patients with hepatocellular carcinoma and renal clear cell carcinoma with or without abdominal radiotherapy (Figure S1). We analyzed the correlation between the abundance of *A. muciniphila* and the duration of diarrhea in patients during a 30-day observation period after the end of abdominal radiation therapy, and found that the abundance of *A. muciniphila* was negatively correlated with the duration of diarrhea in IR patients (Figure 1o). Consistent with the alteration of microbiota in feces from radiotherapy patients, IR also led to changes in the composition of intestinal flora in mice, with a significant reduction in the abundance of *A. muciniphila* (Figure S2).

### Supplement of A. muciniphila mitigates abdominal IR-induced intestinal damage

**Figure 2.**
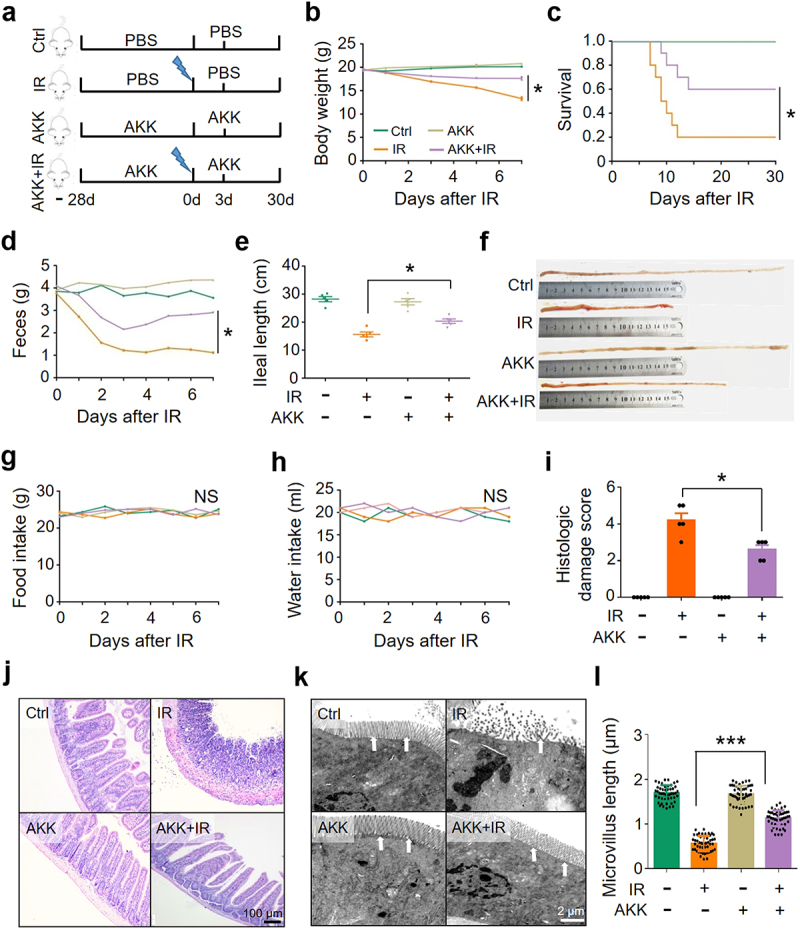
Supplement of *A. muciniphila* mitigates IR-induced intestinal damage.

To determine whether the reduction of *A. muciniphila* plays a role in IR-induced intestinal damage, we pretreated the mice with 1 × 10^8^ CFU of *A. muciniphila* gavage for 28 consecutive days before and 3 days after abdominal IR at 8 Gy. Five mice in each group were euthanized on day 3 post IR for the observation of intestinal damage. Ten mice in each group were used for the observation of murine survival until day 30 post IR (Figure 2a-c). As compared with the application of PBS (control), intragastric administration of *A. muciniphila* markedly increased the abundance of *A. muciniphila* in the intestine (Figure S3a). No changes in the morphology and weight of spleen and the number of peripheral blood cells were observed in abdominal IR mice regardless of the administration of *A. muciniphila*, indicating that abdominal IR did not affect the hematopoiesis of mice (Figure S3b-f). Supplementation of *A. muciniphila* improved the body weight and survival of IR mice as compared with those in PBS-treated IR mice (Figure 2b-c). Fecal weight and ileal length of IR mice remained stable in the exposure to *A. muciniphila* (Figure 2d-f), whereas the intake of food and water in IR mice was not affected (Figure 2g-h).

Healthy BALB/c mice were raised in SPF animal facility. 1 × 10^8^ CFU of *A. muciniphila* in 0.2 mL PBS or PBS alone were intragastrically administered into mice for 4 weeks continuously to re-organize the intestinal microenvironment. The mice were irradiated with 8 Gy abdominal IR or mock treatment. (a) Timeline of the experiment. (b) Body weight of the mice. (c) Survival rate. Ten mice in each group were used for calculating the survival rate in 30 days. (d) Fecal output. (e, f) Ileal length. (g) Food intake. (h) Drinking water intake. (i) Damage score of ileum. (j) HE staining of ileum. (k) Electron microscopy of ileum. Arrow, intestinal microvillus. (l) Intestinal microvillus length (µm) of ileum. Data represent mean ± SEM. * *P* < 0.05, *** *P* < 0.001, compared with PBS-treated IR mice, *n* = 5.

In histology examination and electron microscopy, intestinal epithelial cells (IECs) of IR mice were degenerated, necrotic, and shed. Inflammatory cell infiltration and ileal tissue damage were substantially increased (Figure 2i-l). Congested and swollen crypts and expanded lamina propria were observed. Relative to the treatment of PBS, administration of *A. muciniphila* substantially alleviated the ileal damage (Figure 2i-l), reduced tissue damage score of the intestine in IR mice (Figure 2i). Treatment of *A. muciniphila* maintained the microvillus length and reduced intestinal cell death (Figure 2l). Thus, we conclude that *A. muciniphila* possesses a radiation-protective effect on the intestine of IR mice.

We previously reported that *Lactobacillus* reduces IR-induced intestinal injury^17,18^. *Escherichia coli* (*E. coli*), known as conditional pathogens, were found to negatively regulate IR-induced intestinal injury^19^. We found that, as compared with *E. coli*, *A. muciniphila* and *Lactobacillus* improved the body weight, survival, fecal weight, and ileal length, and reduced intestinal tissue damage of IR mice (Figure S3g-l). In addition, we found that *A. muciniphila* bore a better radioprotective effect against IR-induced intestinal damage in mice than *Lactobacillus* did (Figure S3g-l). To substantiate the radiation-protective effect of *A. muciniphila*, we established an intestinal flora-depleted model of mice by adding 4 antibiotics (ampicillin, metronidazole, neomycin, and vancomycin) in drinking water for 4 weeks^20,21^. Application of the 16S rRNA sequencing, we demonstrated that combination of the 4 antibiotics could effectively deplete intestinal microbiome^18^. We then administered *A. muciniphila* intragastrically to the mice for 4 weeks to reconstitute the microorganisms in the intestinal microenvironment (Figure S4a). The introduction of *A. muciniphila* slowed down the weight loss of mice (Figure S4b) and improved the survival of intestinal flora-depleted mice exposed to abdominal IR (Figure S4c). Consistently, the administration of *A. muciniphila* abrogated the decrease in fecal weight, alleviated the shortening of ileum (Figure S4d-f) and lessened ileal injury in IR mice with intestinal flora-depleted (Figure S4g, h). Collectively, these results support our notion that *A. muciniphila* is critical and sufficient to improve IR-induced intestinal injury and that the effect is *A. muciniphila* specific.

**Figure 3.**
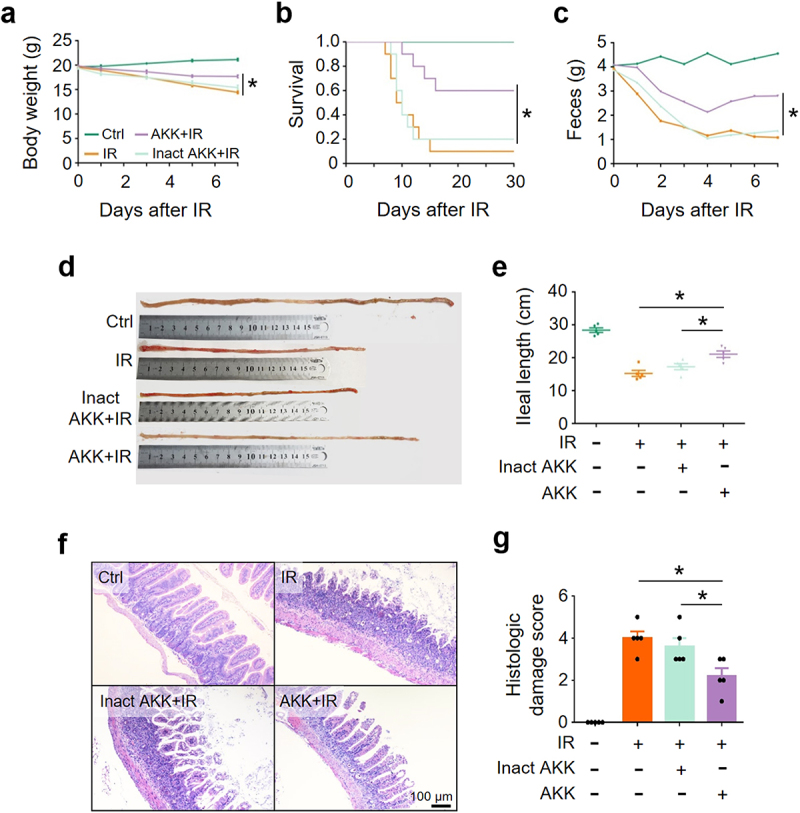
Viable *A. muciniphila* alleviates IR-induced intestinal damage.

To further validate the finding that *A. muciniphila* alleviated IR-induced intestinal damage and promoted the recovery of mice, we compared the outcome of abdominal IR mice treated with live or pasteurization-inactivated *A. muciniphila*. It showed that viable, but not pasteurization-inactivated, *A. muciniphila* ameliorated abdominal IR-induced reduction in murine body weight, fecal volume, and ileal length (Figure 3a-e), alleviated intestinal tissue damage (Figure 3f-g), and promoted murine survival (Figure 3b). Our results indicate that viable *A. muciniphila* mitigates IR-induced intestinal damage, possibly through active substance(s) secreted by the microorganism, rather than the abundance of this bacterium.

Healthy BALB/c mice raised in SPF animal facility were orally supplemented with live (AKK+IR) or pasteurization-inactivated (Inact AKK+IR) *A. muciniphila* for 4 weeks before irradiation with 8 Gy X-ray. (a) Body weight. (b) Survival rate of the mice. Ten mice in each group were used to calculate the survival rate. (c) Fecal output. (d, e) Ileal length. (f) HE staining of ileum. (g) Damage score of ileum. Data represent mean ± SEM. * *P* < 0.05, compared with PBS- or inactivated *A. muciniphila*-treated IR mice, *n* = 5.

### A. muciniphila reduces abdominal IR-induced intestinal damage through its secretion

To assess the possibility that active substances secreted by *A. muciniphila* mediate the radioprotective effect on intestine, we applied the supernatant of *A. muciniphila* culture or the control media to mice by gavage for 28 days followed by abdominal IR (Figure S5a). We found that the body weight, survival, fecal weight, ileal length, and ileal damage score were significantly improved in IR mice treated with supernatant of *A. muciniphila* culture, whereas no protective effects were observed in control media-treated IR mice (Figure S5b-h). In addition, we collected the intestine of mice for organoid culture and treated the intestinal organoids with supernatant of *A. muciniphila* culture. IR treatment markedly reduced the formation of intestinal organoids. The buds and sizes of intestinal organoids were substantially reduced due to the IR. Compared with treatment of control media, pretreatment with the supernatant of *A. muciniphila* culture markedly improved the survival of intestinal organoids exposed to IR. Reductions in the number and size of buds in the intestinal organoids resulted from IR exposure were revoked following the pretreatment of supernatant from *A. muciniphila* culture (Figure S5i, j). Taken together, these results indicate that supernatant from *A. muciniphila* culture promotes the survival of irradiated mice and protects intestinal organoids from IR-induced damage, supporting the notion that *A. muciniphila* plays a role in reducing IR-induced intestinal damage through active substance(s) it secreted.

### A. muciniphila alleviates IR-induced intestinal damage by secreting propionic acid

**Figure 4.**
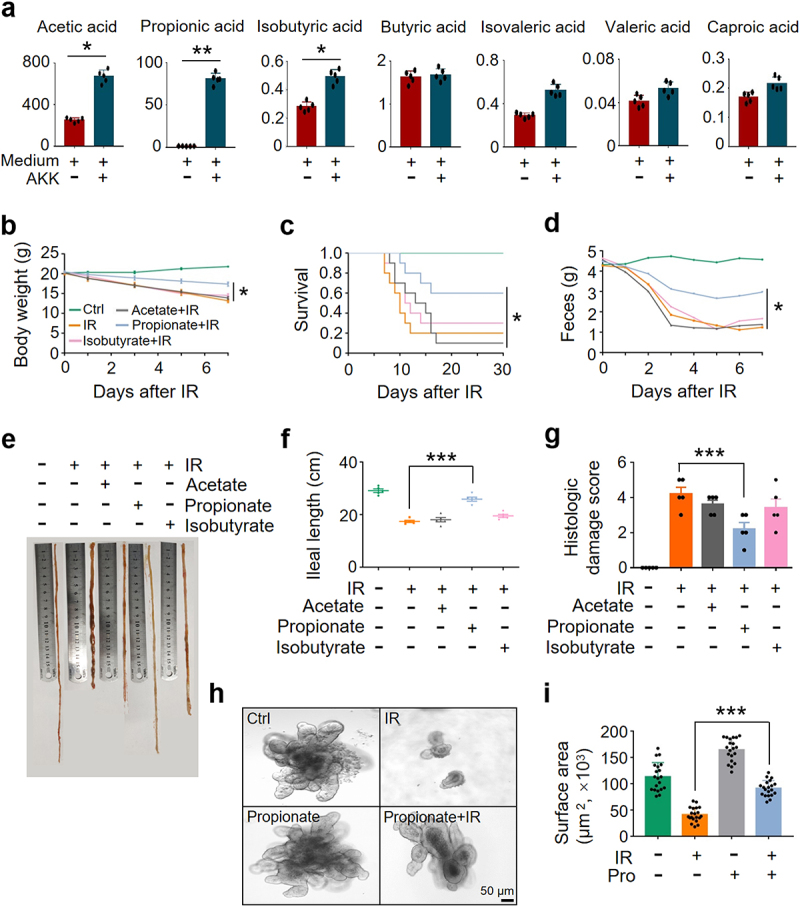
*A. muciniphila* alleviates IR-induced intestinal damage by secreting propionic acid.

*A muciniphila* degrades intestinal mucins to generate SCFAs as bioenergetics sources for itself and for the host, stimulates the colonization of mucus layer flora, and prevents pathogenic bacteria from entering into the mucus layer to destroy IECs^22,23^. To determine whether SCFAs mediate the protective effect of *A. muciniphila* against IR-induced intestinal damage, we analyzed SCFA level in the supernatant of *A. muciniphila* culture and in the sera and intestinal content of mice treated with or without *A. muciniphila* by gas chromatography-mass spectrometry. We found that levels of acetic acid, propionic acid, and isobutyric acid in the supernatant of *A. muciniphila* culture and in the serum and intestinal content of *A. muciniphila*-treated mice were substantially increased, whereas no difference was identified in the levels of butyric acid, isovaleric acid, valeric acid, and caproic acid (Figure 4a and Figure S6).

(a) Supernatants of *A. muciniphila* culture were collected, and levels of SCFAs were measured as described in Materials and Methods. Levels (µg/ml) of acetic acid, propionic acid, isobutyric acid, butyric acid, isovaleric acid, valeric acid, and caproic acid in supernatants of *A. muciniphila* culture were displayed. (b-i) Healthy BALB/c mice raised in SPF animal facility were intragastrically administered with acetate (Sigma, 60 mM), propionate (Sigma, 60 mM), and isobutyrate (Sigma, 60 mM) in 0.2 mL PBS or PBS alone for 4 weeks continuously. (b) Body weight of the mice. (c) Survival rate. (d) Fecal output. (e, f) Ileal length. (g) Damage score of ileum. (h) Intestinal organoid image. (i) Intestinal organoid surface area. Data represent mean ± SEM. * *P* < 0.05, *** *P* < 0.001, compared with PBS-treated IR mice, *n* = 5.

To identify the SCFA(s) secreted by *A. muciniphila* in preventing IR-induced intestinal damage, we evaluated the outcome of acetic acid, propionic acid, and isobutyric acid in the protection of IR-induced intestinal damage in mice. Two hundred µl of 60 mM acetate, propionate, or isobutyrate^24^ were administered to mice by gavage for 28 days before IR. Application of propionate markedly slowed down the weight loss and promoted the survival of IR mice. In contrast, no radioprotective effect was found in mice treated with acetate, isobutyrate, or PBS (Figure 4b-c). Treatment with propionate, but not acetate, isobutyrate, or PBS, led to a substantial increase in fecal weight and ileal length and a reduction in intestinal damage score (Figure 4d-g). Administration of propionate generated a similar radioprotective effect on murine intestine as the gavage of *A. muciniphila* and its culture supernatant did (Figure S7), showing a markedly improved body weight, survival, ileal length, and intestinal damage score (Figure S7). In addition, we collected the intestine of mice for organoid culture. Compared with PBS treatment, pretreatment with propionate markedly improved the number and size of buds in intestinal organoids exposed to IR (Figure 4h-i), similar to those observed in irradiated organoids pretreated with the supernatant of *A. muciniphila* culture (Figure S5i, j). Collectively, our results support the notion that propionic acid is the mediator of *A. muciniphila* in mitigating IR-induced intestinal injury.

### Propionic acid protects intestinal epithelial barrier from IR-induced damage by elevating tight junction proteins and mucin-2

**Figure 5.**
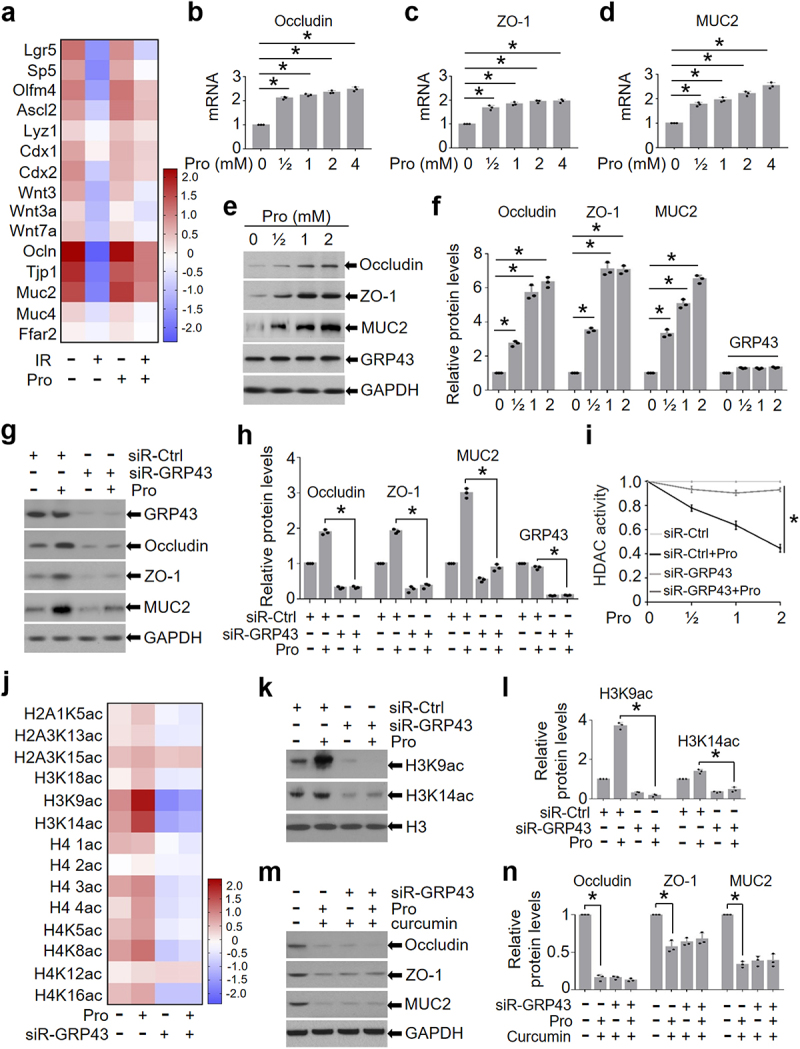
Propionic acid promotes the expression of occludin, ZO-1, and MUC2 through enhancing histone acetylation.

To determine the mechanisms by which propionic acid alleviates IR-induced intestinal injury, we performed an RNA transcriptome sequencing of the intestine from IR mice after propionate treatment. We found that the mRNA levels of occludin (Ocln), ZO-1 (Tjp1), and mucin-2 (Muc2) were increased in the intestine of mice treated with propionate and IR as compared with those in IR mice (Figure 5a). Propionic acid binds to its receptor GRP43/41 on the surface of IECs and hence activates the transcription of tight junction genes^25,26^. Interestingly, transcriptome results showed that the mRNA level of GRP43 (Ffar2) was not altered in the intestine of mice treated with or without propionate in the presence or absence of abdominal IR (Figure 5a), which is consistent with previous reports demonstrating that expression of GRP43 is not affected by its ligands^14,27,28^.

Healthy BALB/c mice raised in SPF animal facility were intragastrically administered with propionate (60 mM) in 0.2 mL PBS or PBS alone for 4 weeks continuously. Intestines from mice were collected. RNAs were isolated from the intestines and subjected to transcriptome sequencing. (a) Heatmap analysis of transcriptome sequencing of the murine intestine. *n* = 5. (b-d) Intestinal organoids of mice were treated with 0 - 4 mM propionate (Pro) for 24 h. Total RNAs of the intestinal organoids were isolated with TRIzol reagents. Levels of occludin, ZO-1, and MUC2 mRNA were determined with qRT-PCR. (e) Expressions of occludin, ZO-1 and MUC2 were detected with western blot. (f) Quantification of (e). (g) Intestinal organoids of mice were transduced with siRNAs against GRP43 or control siRNA for 48 h. The intestinal organoids were then exposed to 2 mM propionate for another 24 h. Intestinal organoids extracts were collected for western blot analysis. GAPDH was detected for loading control. (h) Quantification of (g). (i) HDAC activity of nuclear protein extract after treated with different doses of propionate. (j) Histone proteomics of intestinal organoids prepared in (g). (k) Expressions of H3K9ac and H3K14ac in intestinal organoids prepared in (g). (l) Histograms were generated from the relative band density ratios of histone H3K9ac and H3K14ac to H3. (m) Expressions of occludin, ZO-1 and MUC2 in intestinal organoids treated with curcumin (20 μmol/L) and propionate for 24 h were detected with western blot. (n) Quantification of (m). Data represent mean ± SEM. * *P* < 0.05, repeated 3 times.

To validate the finding that propionate upregulated tight junction molecules and MUC2 in the intestine of IR mice (Figure 5a), we detected the expression of occludin, ZO-1, and MUC2 in intestinal organoids treated with propionate. Application of propionate increased the mRNA and protein levels of occludin, ZO-1, and MUC2 in a dosage-dependent manner (Figure 5b-f). Consistently, the expression of GRP43 (Ffar2) was not affected by propionate (Figure 5e-f).

To assess the necessity of GRP43/41 in propionate-induced upregulation of occludin, ZO-1, and MUC2, we knocked down GRP43 and GRP41 in the intestinal organoids and exposed the organoids to propionate. Depletion of GRP43, rather than GRP41 (Figure S8), markedly reduced the elevation of occludin, ZO-1, and MUC2 in propionate-treated organoids (Figure 5g-h). Taken together, these results suggest that propionic acid increases the expression of tight junction proteins and MUC2 in intestinal epithelia by upregulating their transcription, which is dependent on an intact GRP43.

Next, we tested the impact of propionate on the expression of occludin, ZO-1, and MUC2 in the murine intestine. Abdominal IR led to the destruction of tight junctions in the murine intestine (Figure S9a) and a substantial reduction of occludin, ZO-1, and MUC2 (Figure S9b-h). Application of propionate promoted the maintenance of tight junctions in the intestine of IR mice (Figure S9a) and increased the expression of occludin, ZO-1, and MUC2 (Figure S9b-h). Similarly, administration of *A. muciniphila* elevated the expression of occludin, ZO-1, and MUC2 in the intestine of mice (Figure S10).

Stable expression of occludin, ZO-1, and mucins facilitates intestinal barrier function and promotes the integrity of the intestinal epithelia. Thus, we further assessed the gut barrier function by administering FITC-dextran via gavage to mice 4 h prior to euthanize and measuring the concentration of FITC-dextran in the serum. As depicted in Figure S9i, administration of propionate resulted in a significant reduction of FITC-dextran in the serum of IR mice as compared with that in IR mice without propionate treatment, suggesting an improvement in the gut barrier integrity. Consistently, Periodic Acid-Schiff (PAS) staining, a method used to detect polysaccharides, glycoproteins, glycolipids, and mucins in tissues, showed that treatment with propionate restored the goblet cells in intestine, which was depleted by IR (Figure S9j, k), and maintained the mucus layer on the surface of intestinal villi (Figure S10j, l).

To validate the role of GRP43 in the mediation of propionate-promoted the integrity of the intestinal epithelial barrier, we detected the expression of tight junction proteins and MUC2 in the intestine of IR mice under the treatment with GRP43 regulators. IR mice pretreated with 4-CMTB (10 mg/kg/d, ip), a GRP43 agonist, bore a higher level of occludin, ZO-1, and MUC2 in the intestine of mice in the presence of propionate as compared with those in mice treated with propionate alone (Figure S11a, b). In contrast, pretreatment of GLPG0974 (1 mg/kg/d, oral gavage), a GRP43 inhibitor, abrogated propionate-promoted upregulation of occludin, ZO-1, and MUC2 in the intestine of IR mice (Figure S11c, d), reassuring the necessarity of GRP43 in the mediation of propionic acid-upregulated integrity of intestinal epithelial barrier in IR mice.

### Propionic acid promotes the expression of occludin, ZO-1, and MUC2 by enhancing histone acetylation

One mechanism by which propionic acid regulates downstream targets is to enhance histone acetylation by activation of histone acetyltransferase (HAT) or inhibition of histone deacetylase (HDAC)^29,30^. Given that propionate promoted the expression of tight junction genes and MUC2 in the study (Figure 5), we asked whether propionate upregulates these genes by promoting histone acetylation. To address the question, we treated siR-Ctrl- and siR-GRP43-transduced intestinal organoids with propionate and measured the activity of HDAC. As shown in Figure 5i, propionate treatment significantly reduced the activity of HDAC in siR-Ctrl-transduced organoids, but not in siR-GRP43-transduced organoids (Figure 5i). Histone proteomics analysis showed that propionate treatment enhanced histone acetylation of intestinal organoids, and the most significant changes were observed in H3K9ac and H3K14ac (Figure 5j). Western blot analysis confirmed that propionate treatment elevated the level of H3K9ac and H3K14ac in intestinal organoids, both of which were dependent on GRP43 expression (Figure 5k-l). In addition, we detected the level of histone acetylation in IECs of mice treated with the GRP43 agonist (4-CMTB) or the GRP43 inhibitor (GLPG0974) in the presence or absence of propionate and found that propionate combined with 4-CMTB increased, whereas combination of propionate with GLPG0974 reduced, the level of H3K9ac and H3K14ac in the intestine (Figure S12).

To further validate histone acetylation in the mediation of propionate-induced expression of tight junction proteins and MUC2, we exposed intestinal organoids to 20 μmol/L curcumin, a histone acetyltransferase inhibitor^31^, to reduce histone acetylation. We found that propionate no longer impacted the levels of occludin, ZO-1, and MUC2 in intestinal organoids in the presence of curcumin (Figure 5m-n), emphasizing the importance of histone acetylation in propionate-induced expression of tight junction proteins and mucin. Taken together, our results suggest that enhanced histone acetylation by propionate treatment upregulates the transcription of tight junction proteins and mucins in intestinal epithelia through GRP43, which may eventually be beneficial to the integrity of intestinal epithelia.

### Metformin increases the abundance of A. muciniphila in abdominal IR mice

Metformin is the most widely used drug for the treatment of type 2 diabetes^32,33^. It was reported that metformin promotes glucose sensing in small intestine by regulating gut microbiota^34^. To determine whether metformin plays a role in the maintenance of gut microbiota balance in IR mice, we performed a 16S rRNA sequencing on intestinal flora of abdominal IR mice treated with metformin. We found that metformin improves the composition of gut microbiota in abdominal IR mice and *Lactobacillus* is the most abundant bacteria in the intestine of mice treated with metformin, whereas *A. muciniphila* is the bacterium with the highest fold increase after metformin treatment (Figure S13a-i). In addition, application of metformin mitigated IR-induced intestinal injury (Figure S13j-m) and reduced intestinal damage score in abdominal IR mice (Figure S13n).

**Figure 6.**
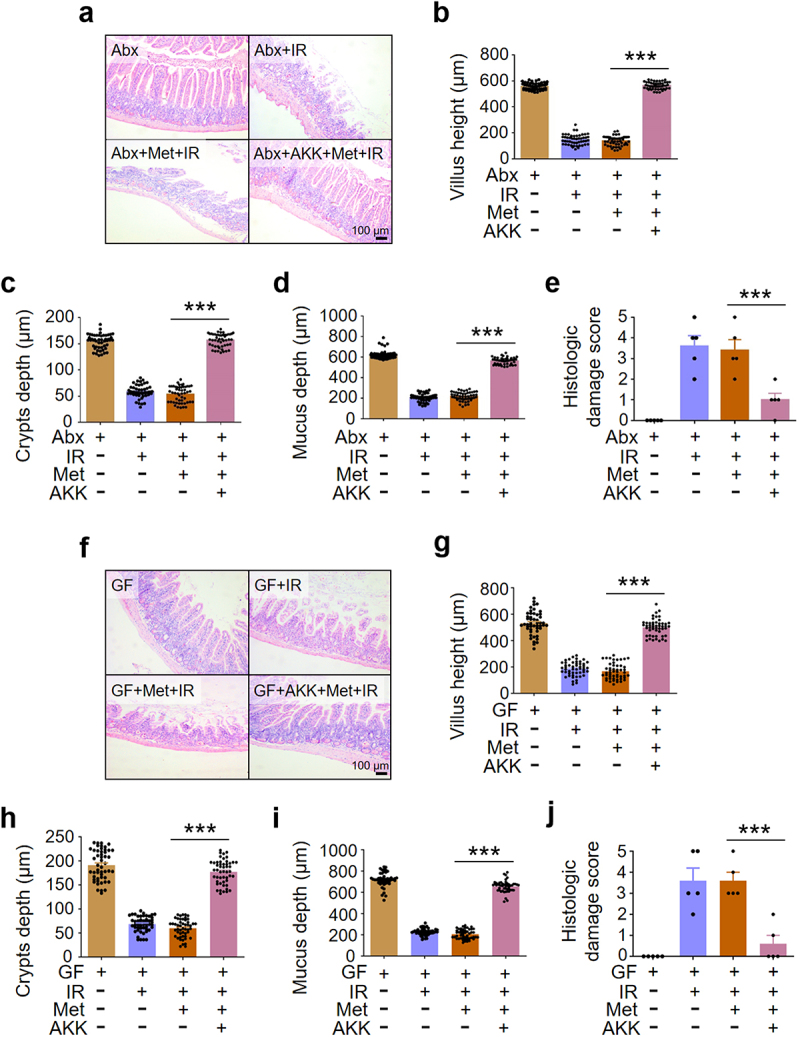
*A. muciniphila* is necessary and sufficient for metformin-induced radioprotection in intestine.

Since *A. muciniphila* was increased with highest fold in metformin-treated mice, we determined the role of *A. muciniphila* in the mediation of metformin-alleviated intestinal damage in IR mice. We reconstituted the intestinal microbiota of microbiota-depleted (Abx) mice and germ-free (GF) mice with *A. muciniphila* and treated the mice with metformin. After reorganization of *A. muciniphila*, administration of metformin increased villus height, crypts depth and mucus depth (Figure 6a-d,f-i) and reduced intestinal damage score in abdominal IR mice (Figure 6e-j), compared with Abx+IR mice and GF+IR mice treated with metformin. Thus, our data demonstrated that *A. muciniphila* was necessary and sufficient for metformin-induced radioprotection in intestine (Figure 6).

Healthy BALB/c mice raised in SPF animal facility were treated with a mixture of 4 antibiotics for 4 weeks. The mice were then orally supplemented with *A. muciniphila* for additional 4 weeks. Metformin was administered continuously for 7 days before and 3 days after IR for a total of 10 days. (a-e) Three days after IR, ilea of microbiota-depleted mice were collected for light microscopy. HE staining (a), intestinal villus height (µm) (b), crypts depth (µm) (c), mucosal layer thickness (µm) (d), and damage score (e). (f-j) Three days after IR, ilea of germ-free (GF) mice were collected for light microscopy. HE staining (f), intestinal villus height (µm) (g), crypts depth (µm) (h), mucosal layer thickness (µm) (i), and damage score (j). Data represent mean ± SEM. *** *P* < 0.001, compared with Abx+Met+IR or GF+Met+IR, *n* = 5.

### Metformin enhances the integrity of intestinal epithelial barrier

**Figure 7.**
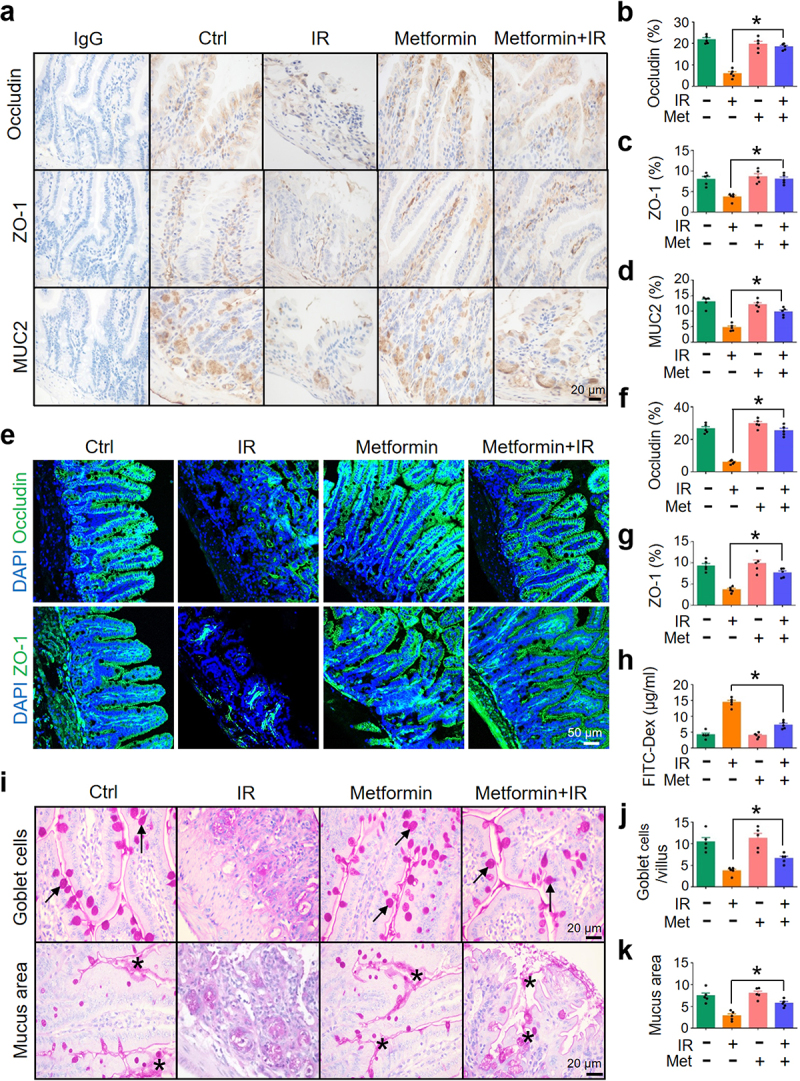
Metformin enhances intestinal barrier function.

To determine whether metformin-reduced intestinal damage in abdominal IR mice is mediated by the maintenance of intestinal epithelial barrier integrity, we detected the level of occludin, ZO-1, and MUC2 in intestinal epithelia in mice treated with metformin. Expressions of occludin, ZO-1, and MUC2 in the intestine of abdominal IR mice treated with metformin were increased as compared with those in IR mice treated with PBS (Figure 7a-g). Consistent with these results, metformin+IR mice exhibited a decrease in serum FITC-dextran levels, a biomarker for intestinal permeability, as compared with IR mice (Figure 7h). The number of goblet cells in the ileum of IR mice was upregulated after metformin treatment (Figure 7i-j). The mucus layer area on the surface of the ileum was also increased (Figure 7i-k). These results suggest that metformin not only increases the abundance of *A. muciniphila* in the intestine, but also enhances the intestinal barrier function and minimizes IR-induced intestinal injury.

Healthy BALB/c mice raised in SPF animal facility were treated with metformin and irradiated with 8 Gy abdominal IR as described in Figure 6. (a) IHC of occludin, ZO-1, and MUC2 in murine ileum. (b-d) Quantitation of occludin (b), ZO-1 (c), and MUC2 (d) in murine ileal epithelium in (a). (e) Immunofluorescence staining of occludin and ZO-1 in murine ileum. (f-g) Quantitation of occludin (f) and ZO-1 (g) in murine ileal epithelium in (e). (h) FITC concentration (μg/ml) in the serum of mice treated with FITC-labeled dextran. (i) PAS staining of goblet cells and mucus in murine intestine. Arrows, goblet cells; Stars, mucus. (j) Goblet cells per villus. (k) Quantitation of the mucus area. Data represent mean ± SEM. * *P* < 0.05, compared with PBS-treated IR mice, *n* = 5.

To assess the role of GRP43 in metformin-maintained the integrity of the intestinal epithelial barrier, we treated the mice with metformin in the presence or absence of GRP43 inhibitor (GLPG0974). Inhibition of GRP43 markedly abrogated the upregulation of occludin, ZO-1, and MUC2 in the intestine of metformin-treated mice (Figure S14a, b). In addition, administration of GLPG0974 also reduced the level of H3K9ac and H3K14ac in the intestine of metformin-treated mice (Figure S14c, d). To further validate histone acetylation in the mediation of metformin-induced expression of tight junction proteins and MUC2, mice were treated with metformin and 20 mg/kg/d curcumin (a histone acetyltransferase inhibitor) by gastric gavages for 7 days before and an additional 3 days after IR for a total of 10 days to suppress histone acetylation. In this case, metformin-induced upregulation of occludin, ZO-1, and MUC2 in the intestine of mice were substantially mitigated due to the co-treatment with curcumin (Figure S14e, f), supporting our notion that histone acetylation mediated metformin-upregulated expression of tight junction proteins and MUC2. Taken together, our results suggest that metformin (and propionate) treatment enhances histone acetylation through GRP43, and hence upregulates the transcription of tight junction proteins and MUC2 in intestinal epithelia, which eventually contributes to the maintenance of integrity of intestinal epithelial barrier.

## Discussion

*A muciniphila* is relatively insensitive to oxygen, and survives under microaerobic conditions to generate additional energy^35^. This feature makes the species in a more advantageous position in the competition with other strict anaerobic bacteria in the intestinal tract. There are at least 12 subspecies of *Akkermansia* colonized in human intestine^22^, among which *A. muciniphila* is the most common and abundant *Akkermansia*^36^. We showed that the diversity and abundance of intestinal flora were decreased in IR-CC patients compared with CC or HC, indicating that IR led to intestinal dysbiosis in patients. The abundance of *A. muciniphila* was negatively correlated with the diarrhea duration of patients. Moreover, in the current study, we successfully established abdominal IR-induced murine intestinal damage model and analyzed microbial diversity of ileal contents using 16S rRNA sequencing. We demonstrated that abdominal IR led to the imbalance of ileal flora toward reduced diversity. *A. muciniphila* was the most severely decreased genus in mice exposed to abdominal IR (Figure S2b). Our investigation showed that the imbalance of intestinal florain particular, the reduction of *A. muciniphila* mediated IR-induced intestinal damage. Administration of *A. muciniphila* alleviated IR-induced intestinal injury and improved the survival of irradiated mice (Figure 2), validating its protective role in mice.

*A muciniphila* prefers to colonize in the mucin-rich intestinal mucus layer, where it specifically degrades mucins to produce SCFAs as carbon, nitrogen, and bioenergetic sources for flora in the intestinal mucus layer and for the host. The whole mucus layer is in a dynamic homeostasis^14^. The outer loose mucus layer in the intestinal cavity is derived from the inner mucus layer and prone to falling off and degradation. The inner mucus layer is replenished by mucins secreted by goblet cells. *A. muciniphila* participates in this dynamic process and plays a critical role in the maintenance of the integrity of the mucus layer (Figure S15). On the one hand, *A. muciniphila* uses mucins as a nutrient source and increased colonization of the bacterium in mucus layer accelerates the mucin degradation^37^. On the other hand, *A. muciniphila* is able to positively regulate the thickness of mucus. It is possible that, it degrades mucins to produce SCFAs, which act as an energy source for host epithelial cells to synthesize and secrete mucus proteins. Furthermore, mucin degradation could stimulate goblet cells to produce more new mucins, which promote the growth and colonization of *A. muciniphila*. Thus, a positive feedback loop maintains continuous renewal of the intestinal mucus layer, rendering a protective role in intestinal epithelial injury (Figure S15). Indeed, our findings support the hypothesis and demonstrate that application of *A. muciniphila* led to increased numbers of goblet cells, promoted the expression of MUC2 in the intestine of IR mice, and extended the mucus layer thickness (Figure S9j-l), which eventually strengthened intestinal epithelial barrier and reduced IR-induced intestinal damage. In short, our data provide strong evidence demonstrating that *A. muciniphila* promotes the integrity of intestinal barrier in humans and animals.

Microbial-generated SCFAs are readily absorbed in the colon, participate in the repair of the wounded epithelium, and regulate glucose and lipid metabolism of the host^14^. Thus, SCFAs exert a protective effect against bacterial pathogens by maintaining the integrity of the epithelial barrier^38^. In the current study, we identified that the function of *A. muciniphila* in the protection of IR-induced intestinal damage is to facilitate the generation of high levels of SCFAs (Figures 4 and S6). We further demonstrated that propionic acid but not acetic acid, butyric acid, or other SCFAs, secreted by *A. muciniphila*, mediated the function of the probiotic. Therefore, *A. muciniphila* may not only play a protective role against bacterial pathogens in physiology but also bear a pathophysiological function in the pathogenesis of certain diseases, such as irradiation-induced intestinal damage (this study) and inflammatory bowel diseases (IBD)^38^.

Recent evidence points to a direct role for the GRP43, GRP41, and GRP109A receptors in mediating the protective roles of SCFAs in IBD^38,39^, as well as in controlling the production of cytokines and chemokines by IECs^38^. GRP43, a G-protein-coupled receptor, is mainly activated by acetic acid and propionic acid^40^. We found that propionate promoted the expression of tight junction proteins occludin and ZO-1 and that GRP43 was necessary for conveying the propionate signal (Figure 5g-h). These tight junction proteins are located in the most apical side of the basolateral membrane, forming the paracellular pathways that regulate the passage of ions, solutes, bacteria, and toxins across the epithelial monolayer and are also responsible for maintaining/imparting cell polarity^41^. The integrity of the tight junction structure largely depends on the expression levels of tight junction proteins^41^. Thus, findings in the current study that *A. muciniphila* upregulates tight junction proteins occludin and ZO-1 through the secretion of propionic acid provide another layer of evidence demonstrating that *A. muciniphila* is critical in the maintenance of the integrity of intestinal epithelial barrier and in the prevention of IR-induced intestinal injury.

Guo et al. identified a population of mice that recovered from high-dose radiation to live normal life spans harbor distinct gut microbiota and metabolites. Commensal-associated propionic acid suppresses radiation-induced death and intestinal and hematopoietic damage of mice^42^. Although propionic acid has individually been reported to decrease intestinal inflammation, it remains unknown how propionic acid alleviates radiation enteritis and if propionic acid plays a role in *A. muciniphila*-mitigated intestinal damage. In this study, we reported that abundance of *A. muciniphila* was markedly reduced in the intestine of mice exposed to abdominal IR and in feces of patients who received abdominal radiotherapy. Analyzing the metabolic products of *A. muciniphila* revealed that propionic acid mediated the radioprotective effect. It is generally believed that SCFAs induce physiological effects by altering the epigenetic state of cells^30^. With a histone proteomics analysis and a following-up validation assay, we found that propionate treatment substantially enhanced histone acetylationin particular, H3K9ac and H3K14ac, which is dependent on an intact expression of GRP43 (Figure 5k-l). Furthermore, we found that propionate no longer upregulated the levels of occludin, ZO-1, and MUC2 in intestinal organoids in the presence of curcumin, a histone acetyltransferase inhibitor, which reduces histone acetylation (Figure 5m-n), implying the importance of histone acetylation in propionic acid-induced expression of tight junction proteins and MUC2. Thus, we propose that *A. muciniphila*-secreted propionic acid acts on its receptor GRP43 and enhances histone acetylation, which leads to the upregulation of tight junction proteins occludin and ZO-1 and eventually contributes to the maintenance of intestinal epithelial barrier integrity. The production of adequate and balanced SCFAs by healthy gut microbiota, such as *A. muciniphila*, is an important factor for the prevention of IR-induced intestinal epithelial barrier damage and avoidance of its resultant damages to the intestine.

*A. muciniphila* is a novel probiotic, which has not been characterized extensively. Interactive microbe in the gut and agents that regulate the abundance of *A. muciniphila* remain largely undefined. Metformin is a first line drug for the treatment of type II diabetes. Reports showed that metformin increases the abundance of gut microbiota in diabetes patients^43^. Metformin was previously reported to reduce radiation-induced lung injury, alleviate pulmonary fibrosis and inflammatory infiltration, and hence bear a potential for the application in radioprotection^44^. Chen et al. reported metformin alleviated radiation intestinal injury by optimizing mitophagy in an AMPK-dependent manner^45^. In the present study, we found that metformin modulated the microbiota composition in the ileum of abdominal IR mice. In particular, *A. muciniphila* in the intestine was markedly elevated by metformin (Figure S13). We reconstituted the intestinal microbiota of microbiota-depleted (Abx) and GF mice with *A. muciniphila* and demonstrated that *A. muciniphila* was necessary and sufficient for metformin-induced radioprotection in the intestine. We further exhibited that application of metformin prior to and post IR reduced the death of IECs and increased intestinal villus height, crypts depth, and mucus layer thickness. In addition, treatment of metformin enhanced the level of tight junction proteins occludin and ZO-1, elevated the number of goblet cells, increased MUC2, and enlarged the mucus layer (Figure S15). Thus, our data endorse a robust role of metformin in the protection of the intestine from IR-induced damage by increasing the abundance of *A. muciniphila* and by enhancing the intestinal barrier integrity.

In summary, in the current study, we identified that *A. muciniphila* was the most severely decreased microorganism in the intestine upon abdominal IR. Administration of *A. muciniphila* to abdominal IR mice substantially mitigated intestinal damage and prevented mice from IR-induced death. We further demonstrated that metformin increased the abundance of *A. muciniphila*, which in turn upregulated the levels of SCFAs in the intestine and in the serum, protected the integrity of intestinal epithelial barrier, and hence mitigated abdominal IR damage. These studies shed new light on the application of probiotics or their regulators, such as metformin, in the protection of radiation exposure-damaged intestine.

## Materials and Methods

### Collection of stool from patients with cervical cancer

We collected feces from cervical cancer patients registered in the Department of Gynecology of Jiangsu Cancer Hospital and underwent abdominal radiotherapy. The irradiation dose was 2 Gy/time, 5 times/week, for 5 consecutive weeks. Inclusion criteria: (1) 30 ~ 60 years old; (2) receiving abdominal radiation therapy; (3) with or without clinical manifestations of radiation-induced intestine injury (tenesmus, diarrhea, rectal bleeding, mucus and fecal incontinence, etc.). Exclusion criteria: radiotherapy was interrupted; inflammatory bowel diseases; tumor involving the bowel; antibiotic administration during radiation therapy; diabetes. Fecal samples from 87 female patients with abdominal IR were collected for 16S rRNA sequencing. In addition, 83 healthy female subjects and 78 female cervical cancer patients without abdominal IR, aged between 30 and 60, were recruited locally, and their stool samples were collected to serve as controls. In addition, we also collected stool samples from hepatocellular carcinoma (*n* = 26 in LIHC and *n* = 27 in IR-LIHC) and renal clear cell carcinoma (*n* = 29 in KIRC and *n* = 23 in IR-KIRC) patients with or without abdominal IR. Informed consent was obtained from the participants for collecting the stool from patients. Patients’ sample collection and use were approved by the ethical committee of Jiangsu Cancer Hospital and Henan University.

### Establishment of radiation injury model in mice

Experiments involving mice were under the regulation of ethical requirements and approved by the institutional animal care and use committee of Henan University in China.

Consistent with the gender of cervical cancer patients, we selected healthy BALB/c female mice for the study. The mice were housed in animal barrier facility in Henan University on a 12 h light-dark cycle with food and water available *ad libitum*. Mice were randomly assigned into cages for control or IR treatment in the presence or absence of metformin, or for the collection of feces. Eight Gy one-time abdominal IR with a dose rate of 1.0 Gy/min by X-Ray was applied^46^. Five mice in each group were euthanized on day 3 post IR for the observation of intestinal damage. Ten mice in each group were used for monitoring mouse survival until day 30 post IR. Each mouse was fed and housed separately and data were generalized from single cage. Each experiment was repeated at least 3 times to ensure the stability and repeatability of the experiment.

Metformin was intragastrically administered at a dose of 250 mg/kg/d^47^. Mice were treated with metformin continuously for 7 days before and additional 3 days after IR for a total of 10 days.

### Collection of feces and ileal contents from mice

Fresh feces from mice were collected after IR. Thirty mg of feces were kept in sterilized Eppendorf tubes and maintained in −80°C. After the mice were euthanized, ileocecal area in abdominal cavity was located. Sixty mg of mucosa and intestinal content within 5 cm-distal ileum were collected by scraping^18^.

### 16S rRNA sequencing

The sequencing was performed by using the Illumina Hiseq platform (Meiji Biology Company, Shanghai, China). DNAs in the intestinal contents of mice were extracted with a DNA extraction kit (GenElute™ Stool DNA Isolation Kit, Sigma-Aldrich), and the purity and concentration of DNA extracted were detected by a ultraviolet spectrophotometer and subjected to 1% agarose gel electrophoresis to determine the integrity of DNA. After DNA was qualified, PCR amplification was carried out, and 16S rRNA gene V3-V4 region was amplified. PCR amplification primers for V3-V4 region were 338 F (ACTCCTACGGGAGGCAGCAG) and 806 R (GGACTACHVGGGTWTCTAAT)^48^. The amplified sample was visualized by 2% agarose gel electrophoresis. PCR products at target band size were subject to high-throughput sequencing using the Illumina Hiseq platform (Meiji Biology Company, China). Sequences analysis was performed through Uparse software. ≥97% similarity of sequences were classified to the same OTUs (operational taxonomic units). A representative sequence for each OTU was selected for annotation. For each representative sequence, the Silva123 database was applied to annotate taxonomic information according to the RDP classifier algorithm.

### Quantitative detection of short chain fatty acids (SCFAs) by gas chromatography – mass spectrometry (GC-MS)

Both the qualitation and quantitation of SCFAs were processed by Metware Biotechnology Co., Ltd (Wuhan, China). The measurement was performed following the protocol previously reported^49^. Briefly, Agilent 7890B gas chromatograph coupled to a 7000D mass spectrometer was applied for the detection. A DB-5 MS column was used for the detection. Helium was used as carrier gas, at a flow rate of 1.2 mL/min. Injections were made in the splitless mode and the injection volume was 2 μL. Samples were analyzed in multiple reaction monitoring mode. SCFAs mixtures were prepared to construct the calibration curves.

### Intestinal organoid culture

6–8 week-old BALB/C mice were euthanized by decapitation. 10 cm of terminal ileum was taken immediately and washed with pre-cooled (4°C) PBS. Adipose tissue and mesentery in the intestine were removed. Intestinal contents were washed away with 4°C pre-chilled PBS. After the ileum was cut into 2 mm pieces, pipetting was repeated 6 times with PBS. The supernatant was removed after the tissue was precipitated. EDTA-containing PBS was added to precipitates and incubated for 30 min at 4°C on a shaker. After incubation, pipetting was repeated 6 times with PBS until the crypts are dispersed. 200–500 crypts per well were suspended in Matrigel (Corning). Complete ENR medium (all components from Thermo Fisher Scientific) consisted of advanced DMEM/F12 (Gibco), antibiotic and antifungal (×100), 1 mM N-acetylcysteine (Sigma-Aldrich), B27 Supplement, N2 Supplement, EGF, Noggin (R&D Systems), R-spondin-1-conditioned medium was added for the culture. The ENR medium was replaced every 2 to 3 days. The surface area of intestinal organoids was measured from non-overlapping photographs of the organoids randomly taken in a well using an inverted microscope (Carl Zeiss). Each photo was analyzed using ImageJ software (NIH) and the Zen image program (Carl Zeiss)^50^.

### Hematoxylin-eosin (HE) staining and immunohistochemistry (IHC)

The paraffin sections were placed in xylene for overnight. Complete dewaxing with fresh xylene and graded alcohol hydration were carried out on the following day. The slides were then stained with HE^51^. Additional sections were used to probe the expression of occludin, ZO-1, and MUC2 in ilea by IHC using antibodies from Abcam (USA) following thermal antigen retrieval. Endogenous peroxidase blocking, antibody action, and development were performed as reported^51^. Quantification of positive areas in intestinal epithelial cells was completed using IHC plugin of ImageJ software^52,53^.

### Immunofluorescence

Ileal tissues from mice were snap-frozen for immunostaining. Tissue sections were fixed with 4% paraformaldehyde in PBS for 30 minutes, followed by blocking with 5% normal goat serum (NGS) for 2 h. Antibodies against ZO-1 (Abcam, USA) and occludin (Abcam, USA) were incubated at 1:100 dilution in PBS with 1% NGS and incubated overnight at 4°C. The sections were mounted using Slowfade Gold antifade with DAPI reagent (Invitrogen), followed by imaging with LSM 8000 laser confocal microscope (Carl Zeiss AG).

### Histone proteomics

Cells were trypsinized, washed once with PBS, and then histones were extracted and derivatized. Derivatized histone peptides were injected into a Dionex Ultimate 3000 nanoflow HPLC using a Waters nanoAcquity UPLC C18 column (100 m × 150 mm, 3 m) and connected to a Thermo Fisher Q-Exactive mass spectrometer at 700 nl/min. UPLC MS analysis was then performed as described by Thomas et al.^30^. Data were collected using data independent acquisition (DIA) mode.

### Electron microscopy

The mouse ilea were rinsed with physiological saline, rapidly immersed in glutaraldehyde solution (4%) for pre-fixation. The tissues were then subjected to TEM biological sample preparation to complete the sample rinsing, post-fixation, rinsing, block dyeing, gradient dehydration, infiltration, embedding, ultra-thin slicer sectioning, positive staining, and electron microscopy observation^54^.

### Periodic acid-schiff (PAS) staining

The paraffin sections were dewaxed and hydrated as described above. The slides were stained with 1% periodic acid for 10 minutes. After washing thoroughly with distilled water, the slides were stained with Schiff reagent for 30 minutes. Slides were then rinsed in lukewarm tap water, counterstained with HE, dehydrated, and mounted with a synthetic mount medium. The area of mucus in the intestine was measured by taking 5 random non-overlapping photographs of the intestine using a light microscope (*n* = 5 per group)^55^. Each photo was analyzed using ImageJ software (NIH) and the Zen image program (Carl Zeiss) for calculating the area of mucus.

### Histological injury score

Intestinal epithelial injury was classified using the Chiu’s method^56:^ 0, normal intestinal mucosal villi; 1, capillary hyperemia and cystic gaps under the epithelium at the villus apex; 2, cystic gaps enlarged under the epithelium, edema extended in the lamina propria, and central cheliferous vessels dilated; 3, degeneration and necrosis of IECs, severe edema in the lamina propria, and rarely seen abscission in villus apexes; 4, degeneration, necrosis, and exfoliation of IECs, hyperemia, dilation of capillary, uncovering of lamina propria, abscission in some villi, and 5, bleeding, ulceration, disintegration of the lamina propria, and abscission of villi^57^.

### FITC dextran assay

Fluorescein isothiocyanate (FITC)-labeled dextran (FD4, Sigma-Aldrich, USA) was used for the measurement of gut mucosal permeability as described previously^58^. PBS-diluted FITC-dextran (500 mg/kg) was administered to mice via oral gavage. Mice were euthanized after 4 h, and serum was collected by cardiac puncture, centrifuging at 10,000 g for 10 min. The FITC-dextran concentration in the serum was determined based on a standard calibration curve.

### Establishment of intestinal microbiota-depleted mice

Mice were treated with a mixture of four antibiotics in drinking water for 4 weeks^59^. The antibiotic mixture consists of ampicillin (200 mg/L), metronidazole (200 mg/L), neomycin (200 mg/L), and vancomycin (100 mg/L), which were purchased from MedChemExpress, China. The presence of microbes in the feces of mice was determined by 16S rRNA.

### Culture and pasteurization of A. muciniphila

*A. muciniphila* was purchased from the American Type Culture Collection (BAA-835) and cultured in brain heart infusion media (BD Bioscience) supplemented with 0.4% mucin (Sigma) under strict anaerobic conditions at 37°C^60^. Cultures were centrifuged and the culture pellet was suspended in PBS. 1 × 10^8^ CFU of *A. muciniphila* in 0.2 mL PBS or PBS alone was intragastrically administered into mice for 4 weeks continuously to re-organize the intestinal microenvironment before IR. In separate experiments, aliquots of *A. muciniphila* culture were inactivated by pasteurization for 30 min at 70°C^60^. No viable *A. muciniphila* could be recovered. Administration of active or inactivated *A. muciniphila* was conducted as described.

### Statistical analysis

SPSS 17.0 was used for statistical analysis of experimental data. One-way ANOVA and t-test were used for the significant test. *P* < 0.05 (* *P* < 0.05, ** *P* < 0.01, *** *P* < 0.001) was defined as statistically significant, whereas *P* > 0.05, the experimental results were set to be statistically insignificant.

## Supplementary Material

Supplemental Material

## Data Availability

The authors declare that all the data supporting our findings in the study are available within the paper and its supplementary information files.

## Abbreviations

*A. muciniphila*: *Akkermansia muciniphila*
Abx: microbiota-depleted
COX-2: cyclooxygenase-2
GRP: G-protein coupled receptor
IBD: inflammatory bowel disease
IHC: immunohistochemistry
IR: irradiation
MUC2: mucin 2
PAS: Periodic Acid-Schiff
SCFAs: short-chain fatty acids
TLR-2: toll like receptor 2
ZO-1: zonula occludens-1
