# Supplementary figures and images for: Akkermansia muciniphila protects the intestine from irradiation-induced injury by secretion of propionic acid
Source: Gut Microbes. 2023 Dec 12;15(2):2293312. doi: 10.1080/19490976.2023.2293312 (PMC10730217; doi:10.1080/19490976.2023.2293312)

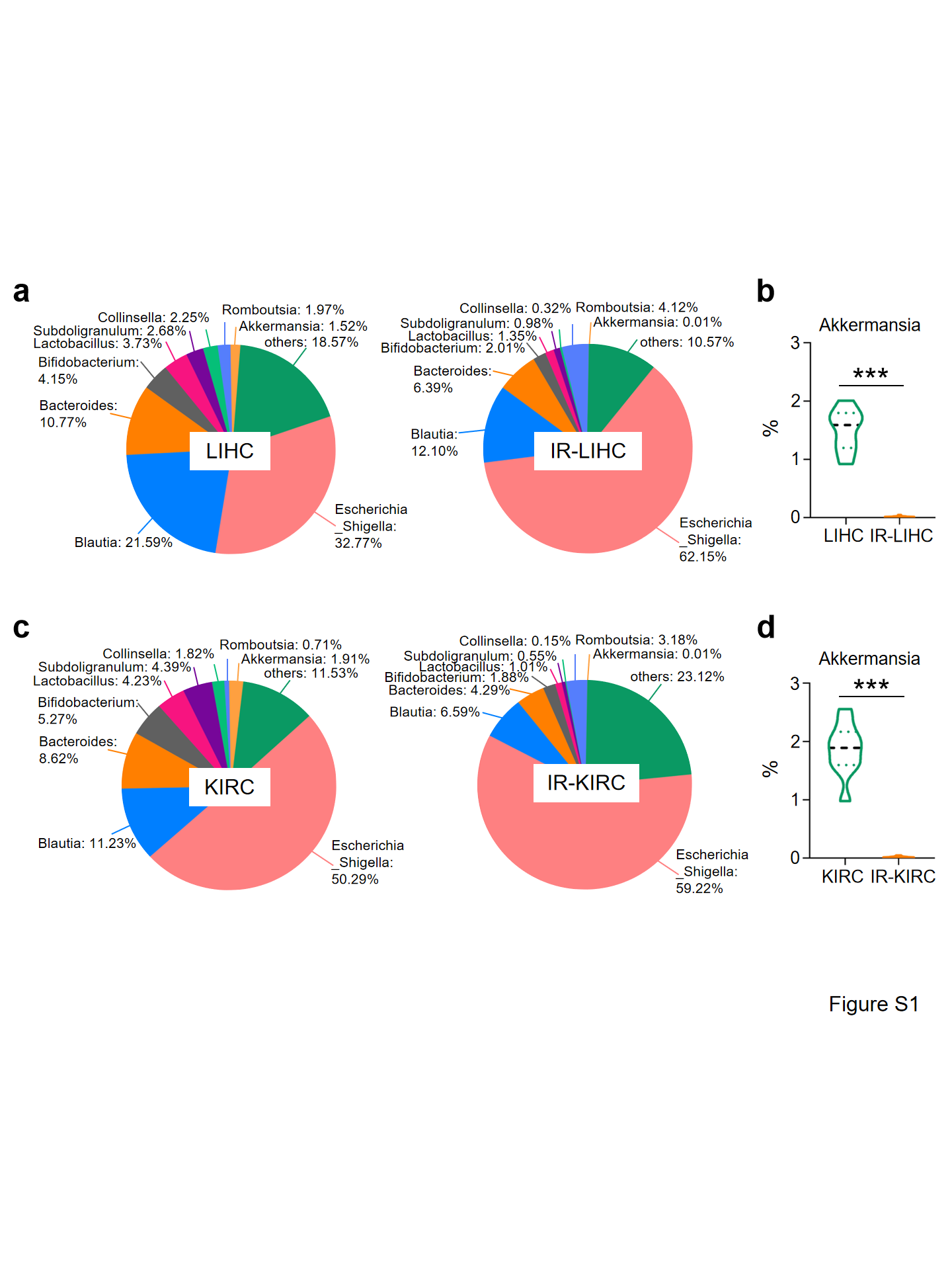

Supplement: Supplemental Material [file KGMI_A_2293312_SM0132.zip › Figure S1 (1).tif]

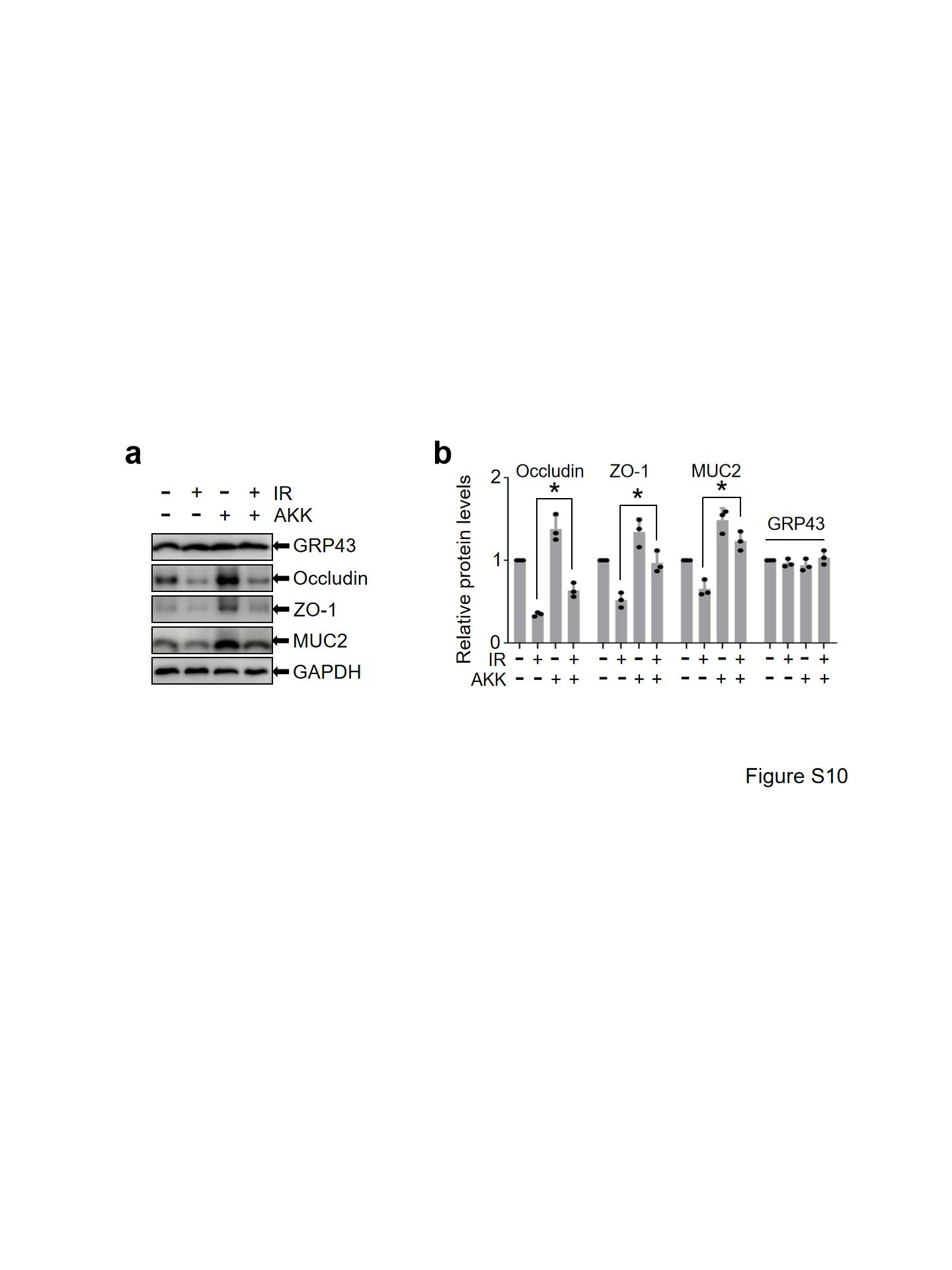

Supplement: Supplemental Material [file KGMI_A_2293312_SM0132.zip › Figure S10.tif]

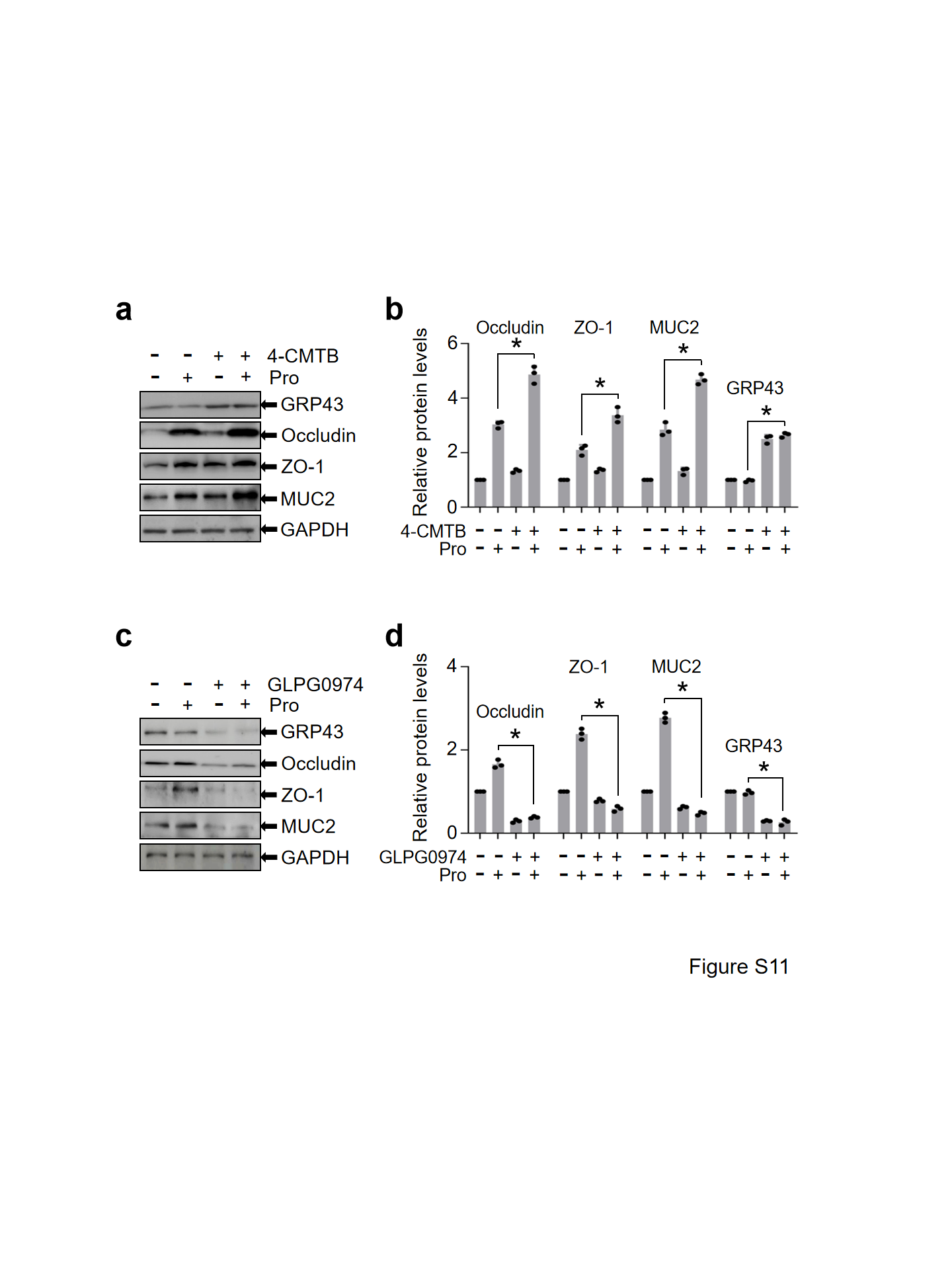

Supplement: Supplemental Material [file KGMI_A_2293312_SM0132.zip › Figure S11.tif]

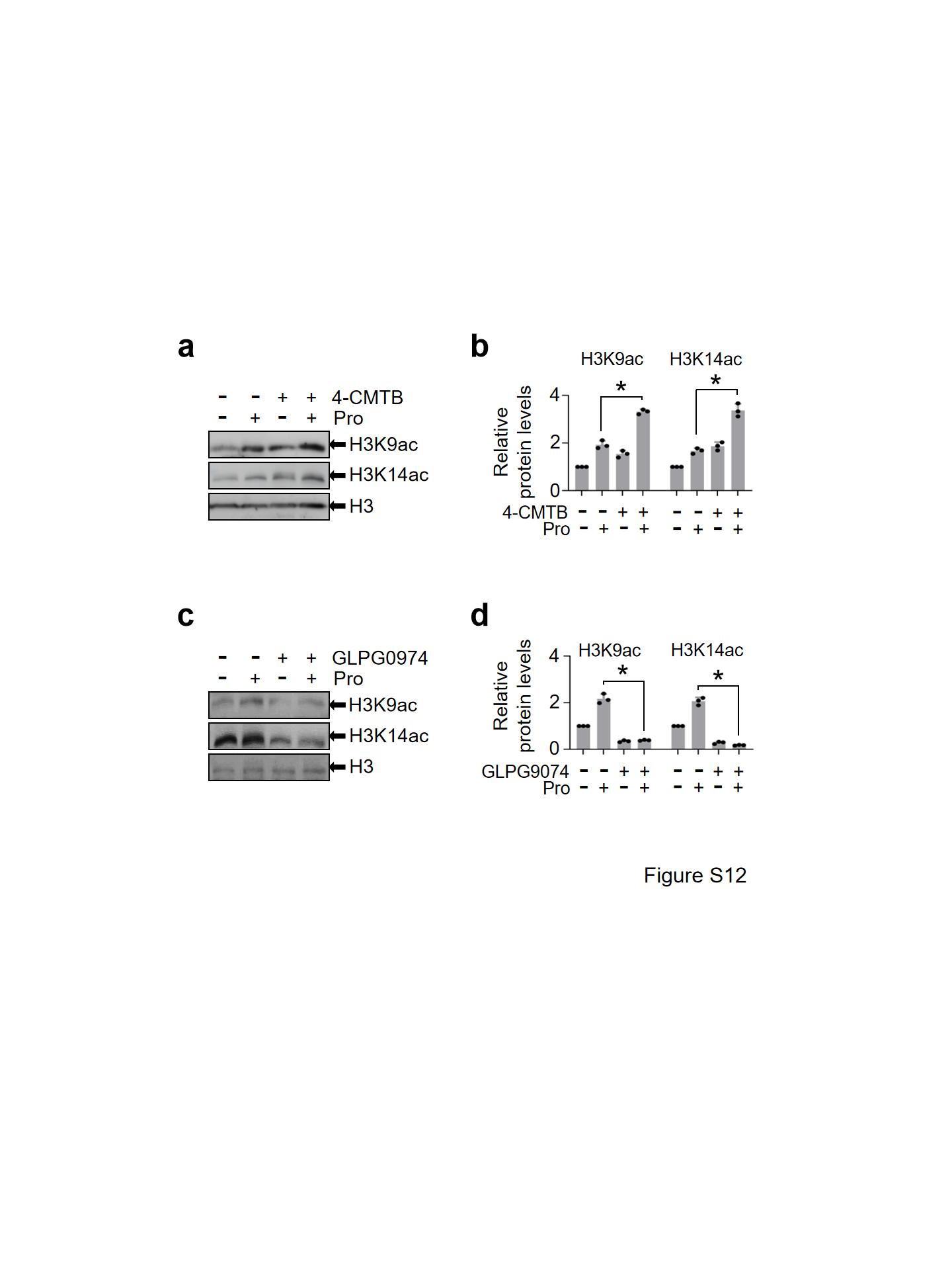

Supplement: Supplemental Material [file KGMI_A_2293312_SM0132.zip › Figure S12.tif]

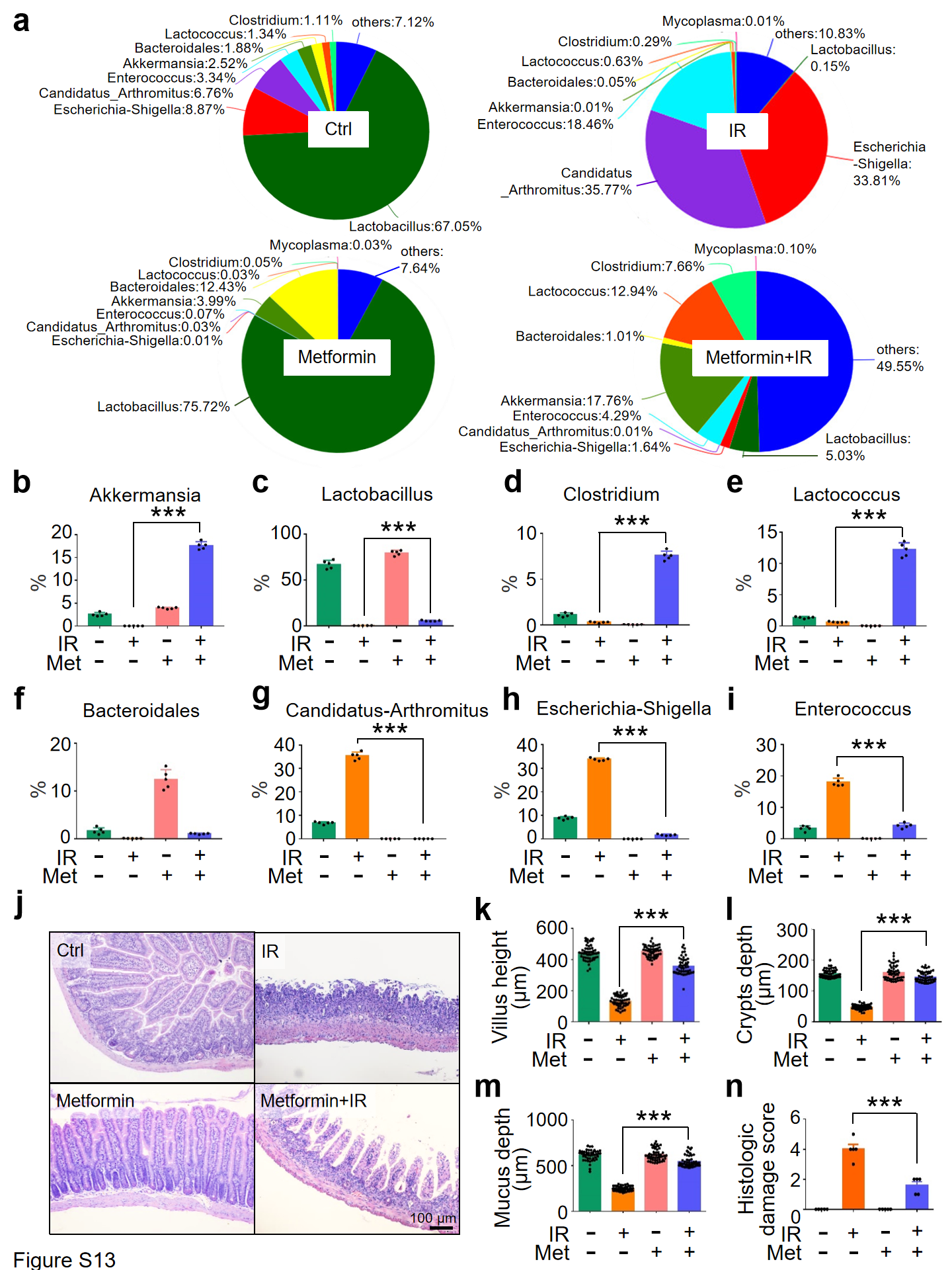

Supplement: Supplemental Material [file KGMI_A_2293312_SM0132.zip › Figure S13.tif]

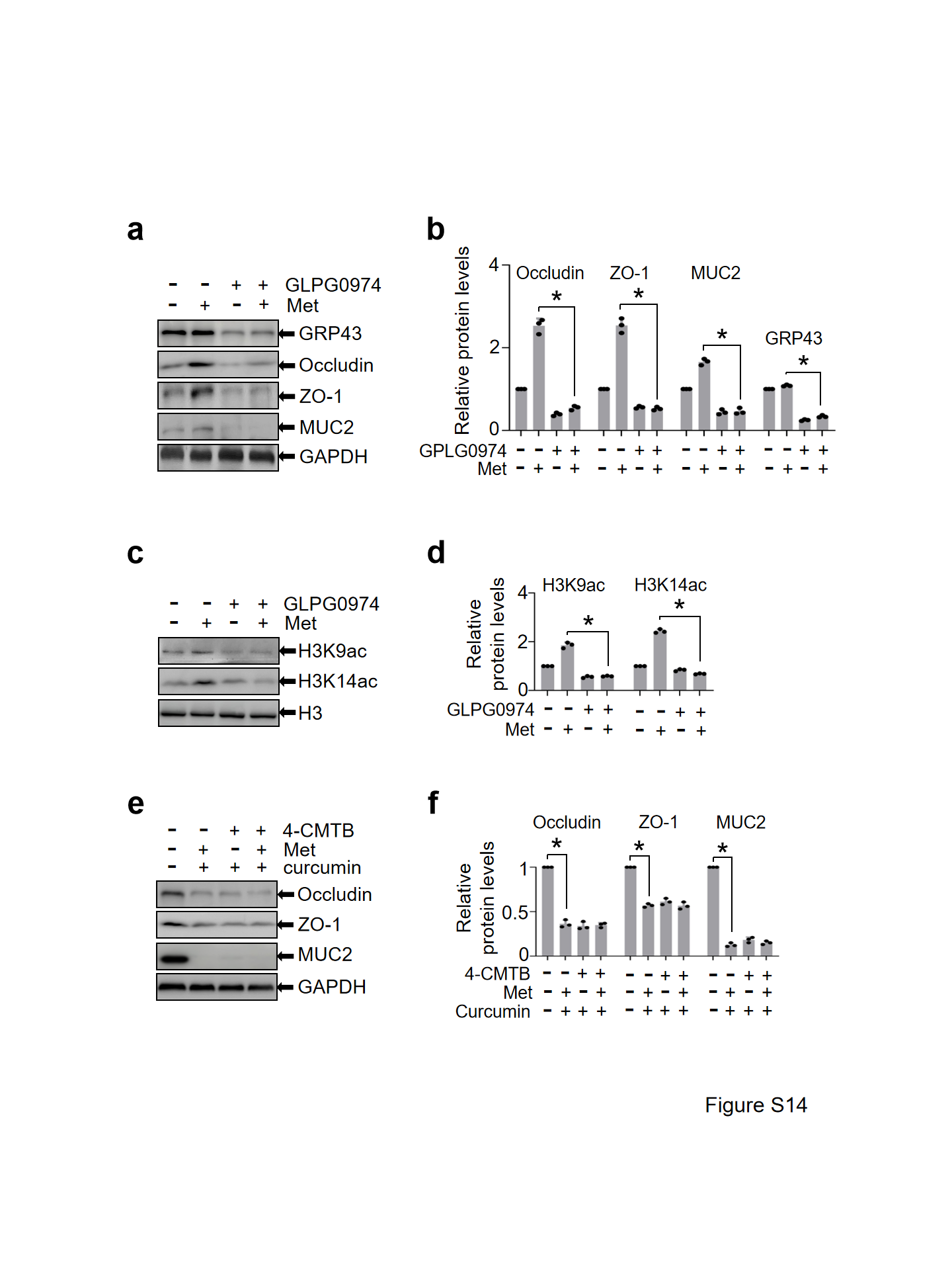

Supplement: Supplemental Material [file KGMI_A_2293312_SM0132.zip › Figure S14.tif]

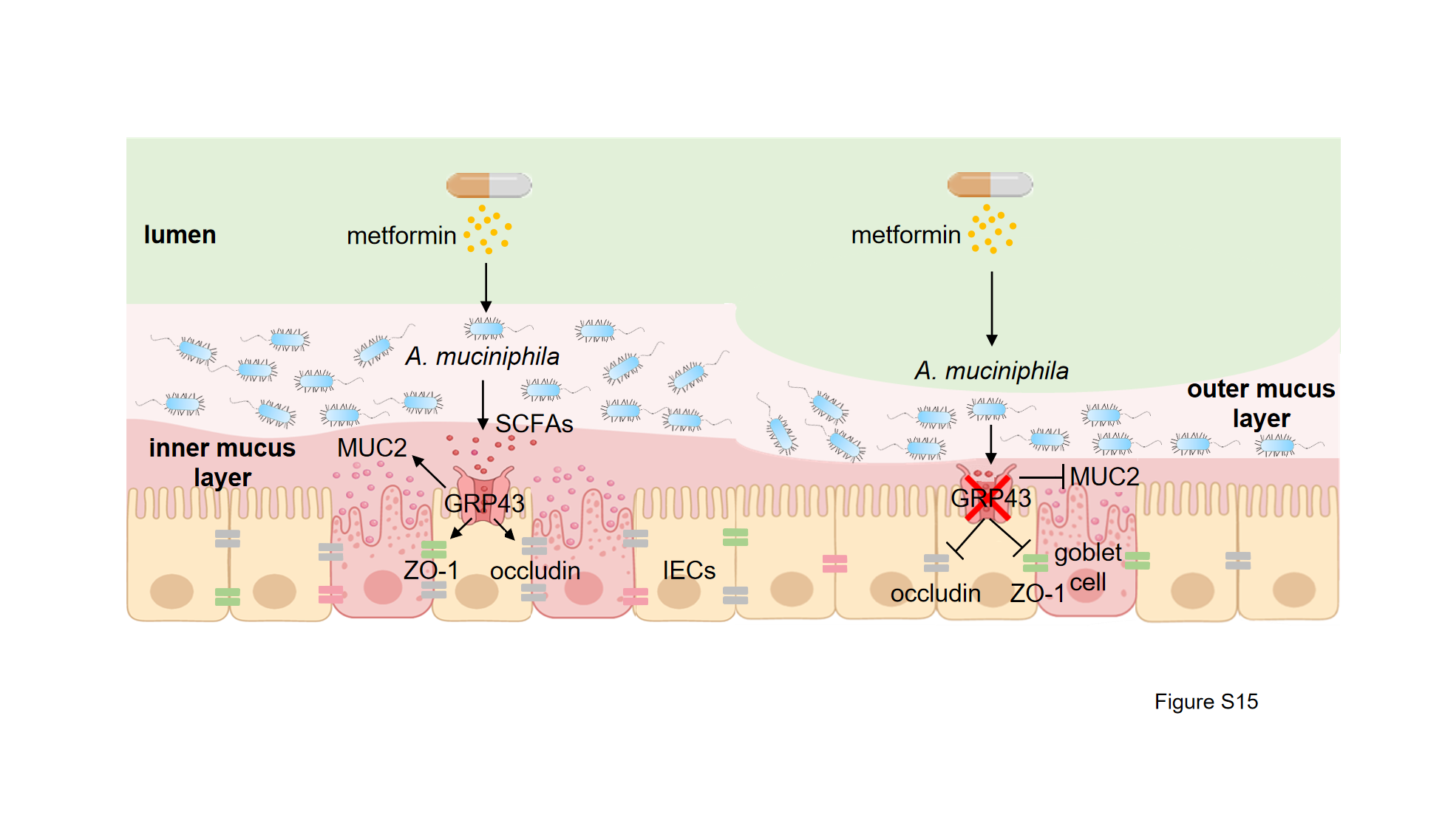

Supplement: Supplemental Material [file KGMI_A_2293312_SM0132.zip › Figure S15.tif]

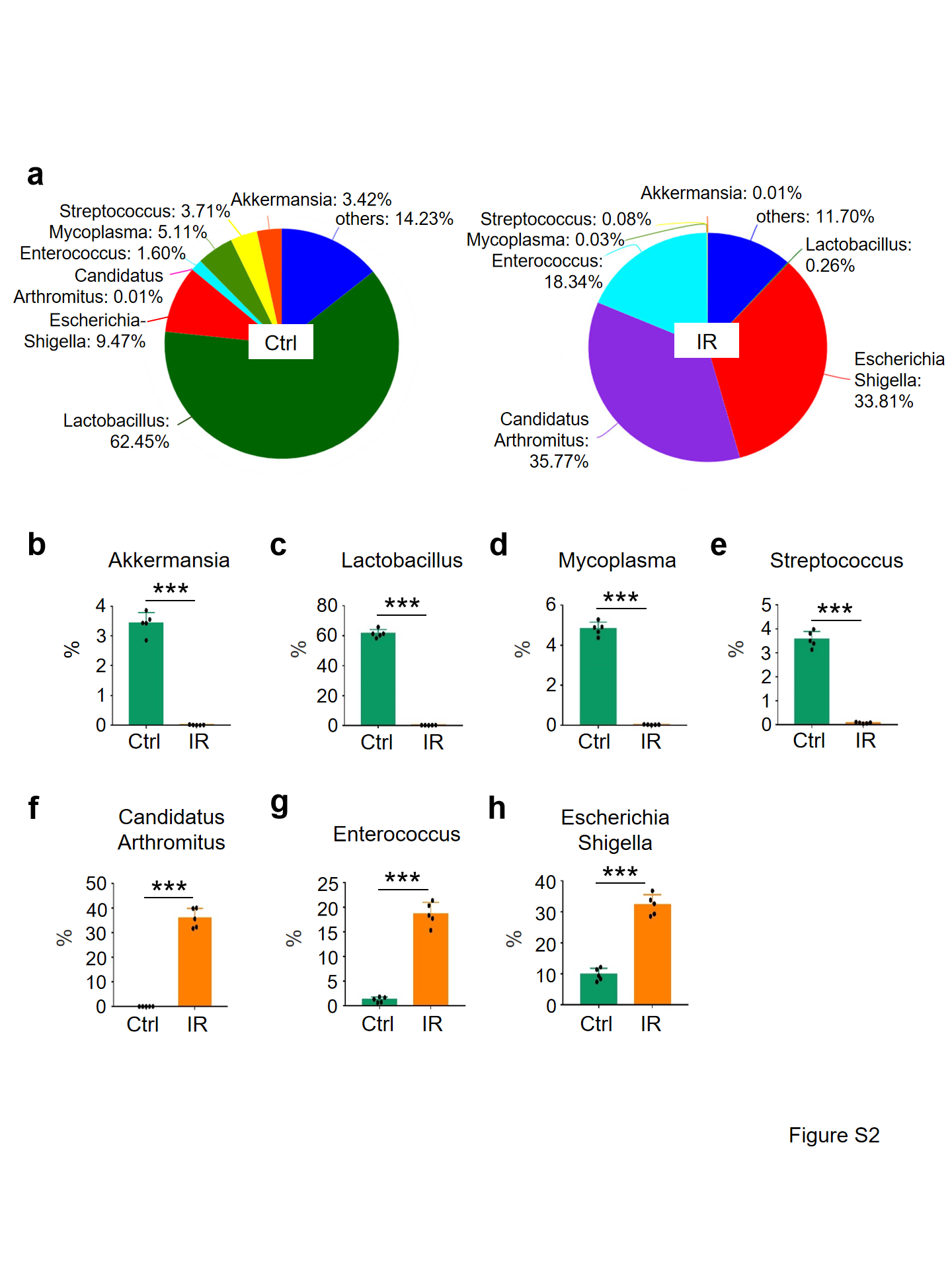

Supplement: Supplemental Material [file KGMI_A_2293312_SM0132.zip › Figure S2 (1).tif]

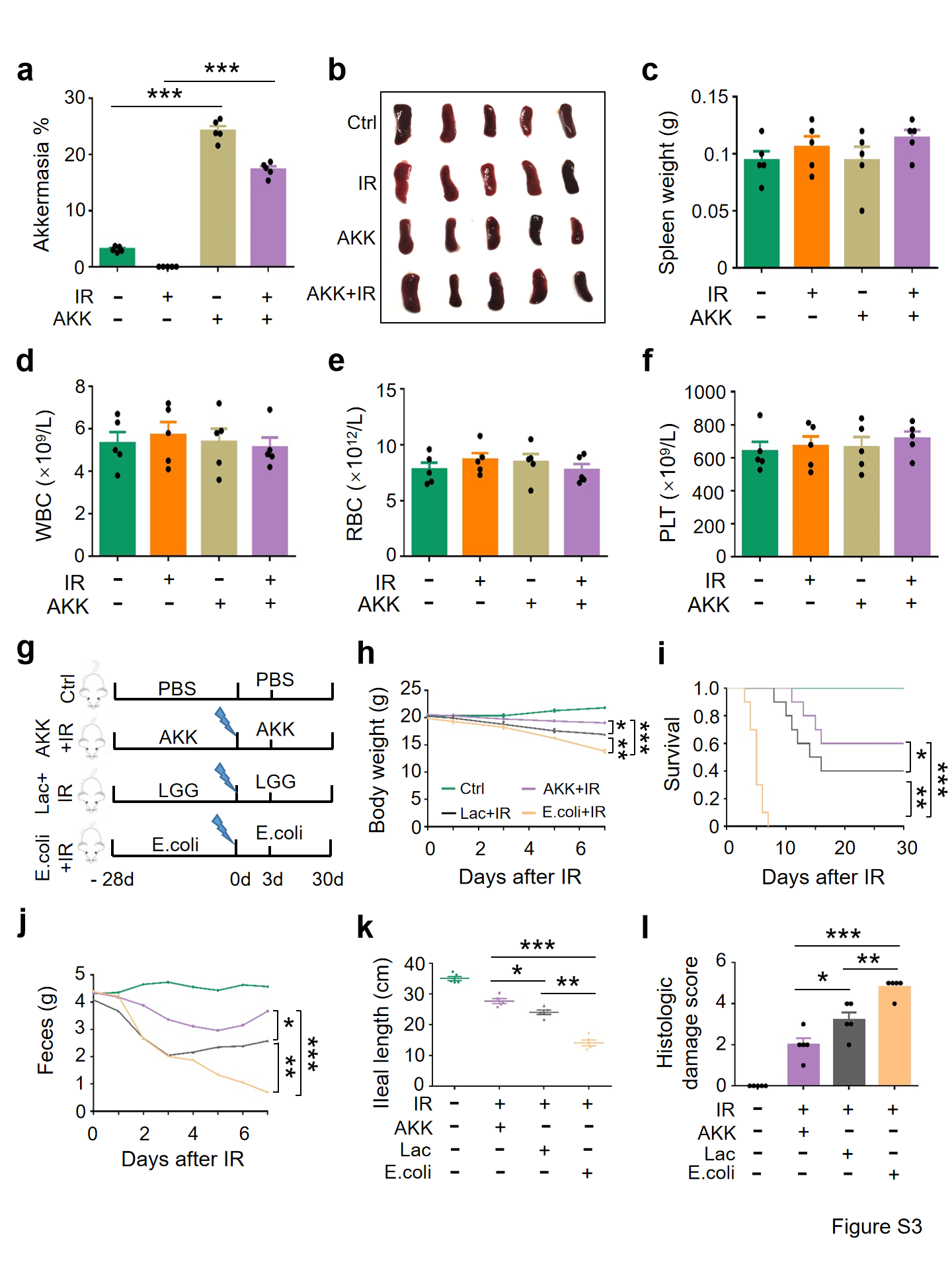

Supplement: Supplemental Material [file KGMI_A_2293312_SM0132.zip › Figure S3 (1).tif]

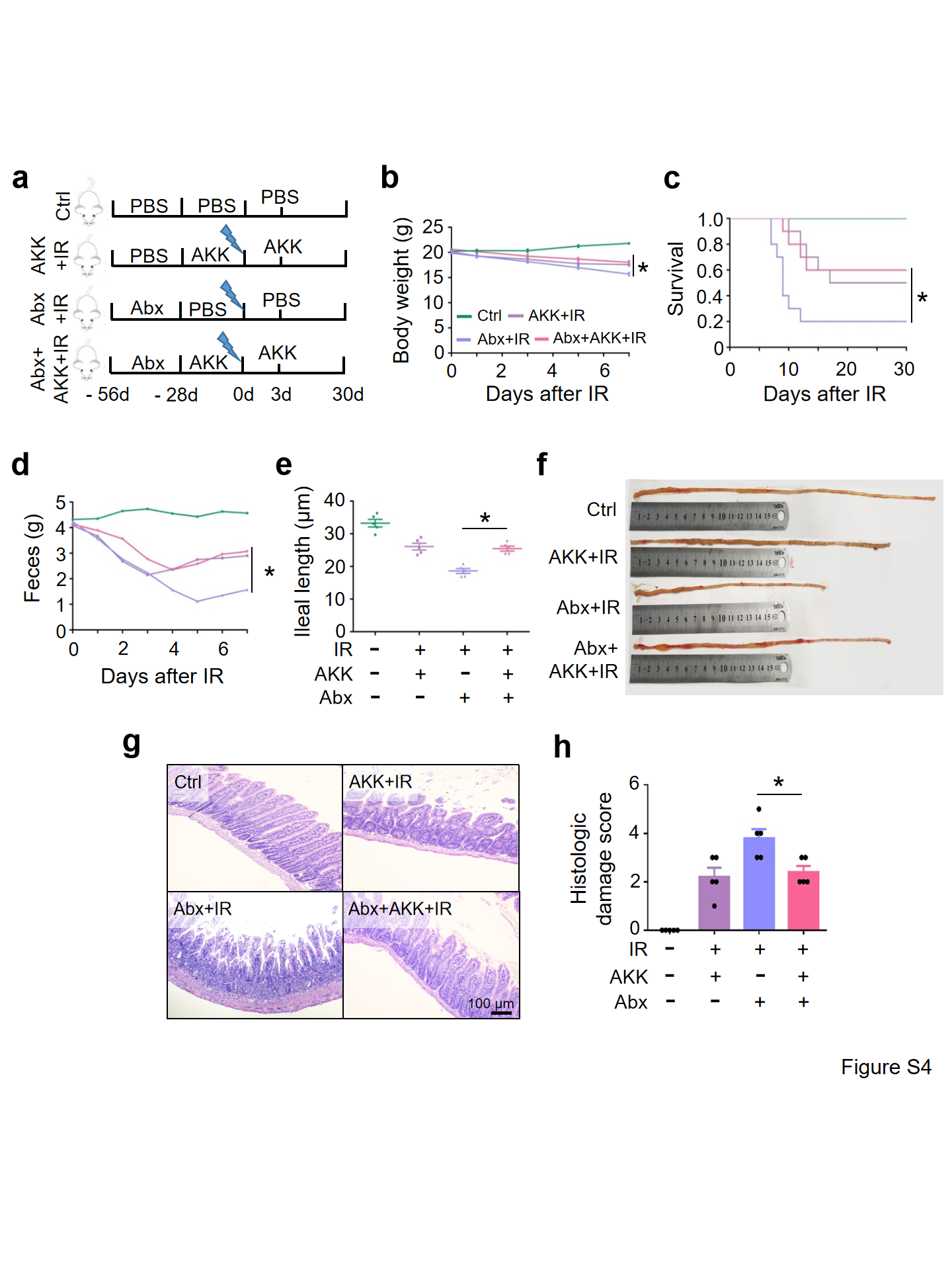

Supplement: Supplemental Material [file KGMI_A_2293312_SM0132.zip › Figure S4 (1).tif]

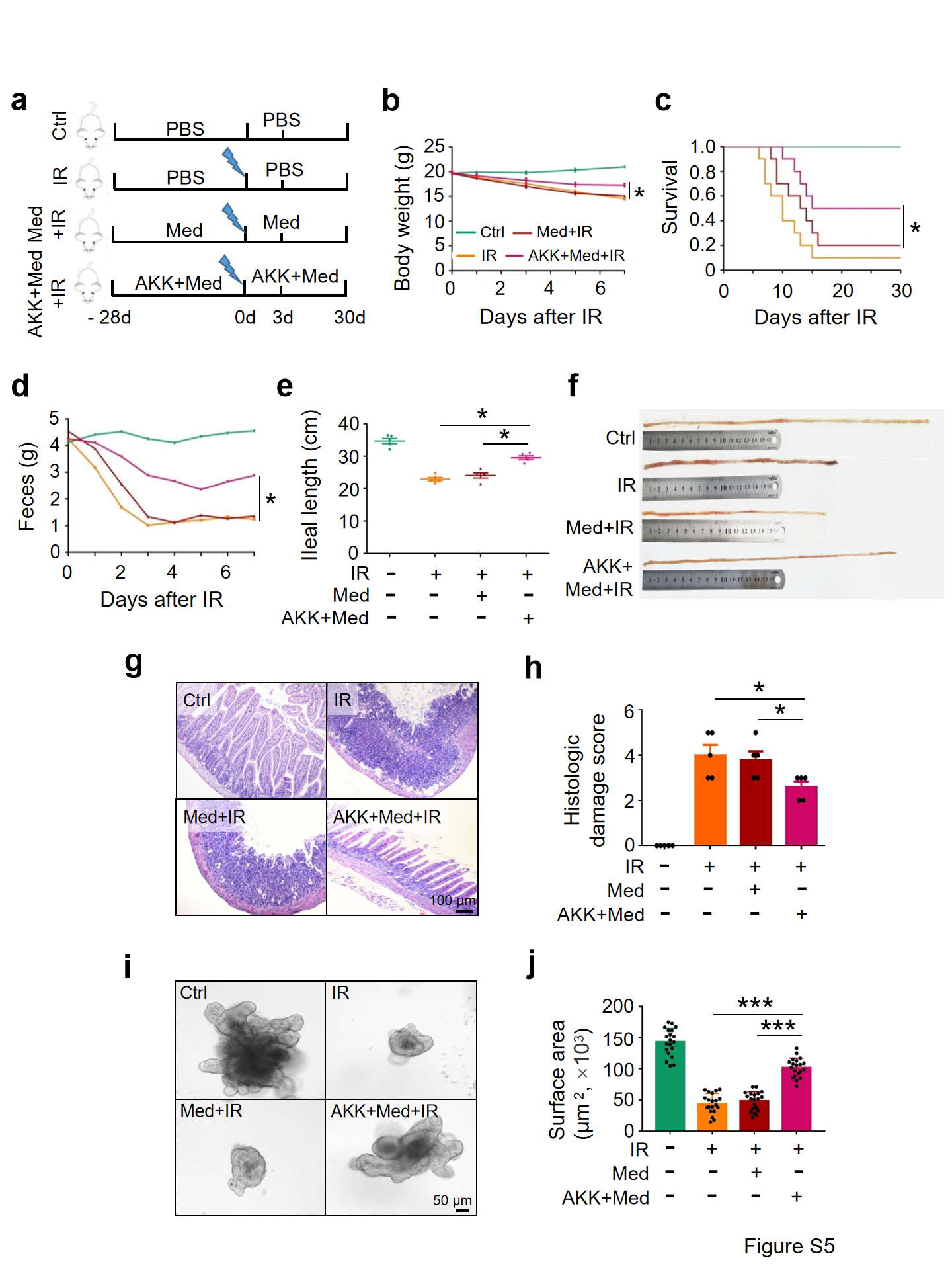

Supplement: Supplemental Material [file KGMI_A_2293312_SM0132.zip › Figure S5 (1).tif]

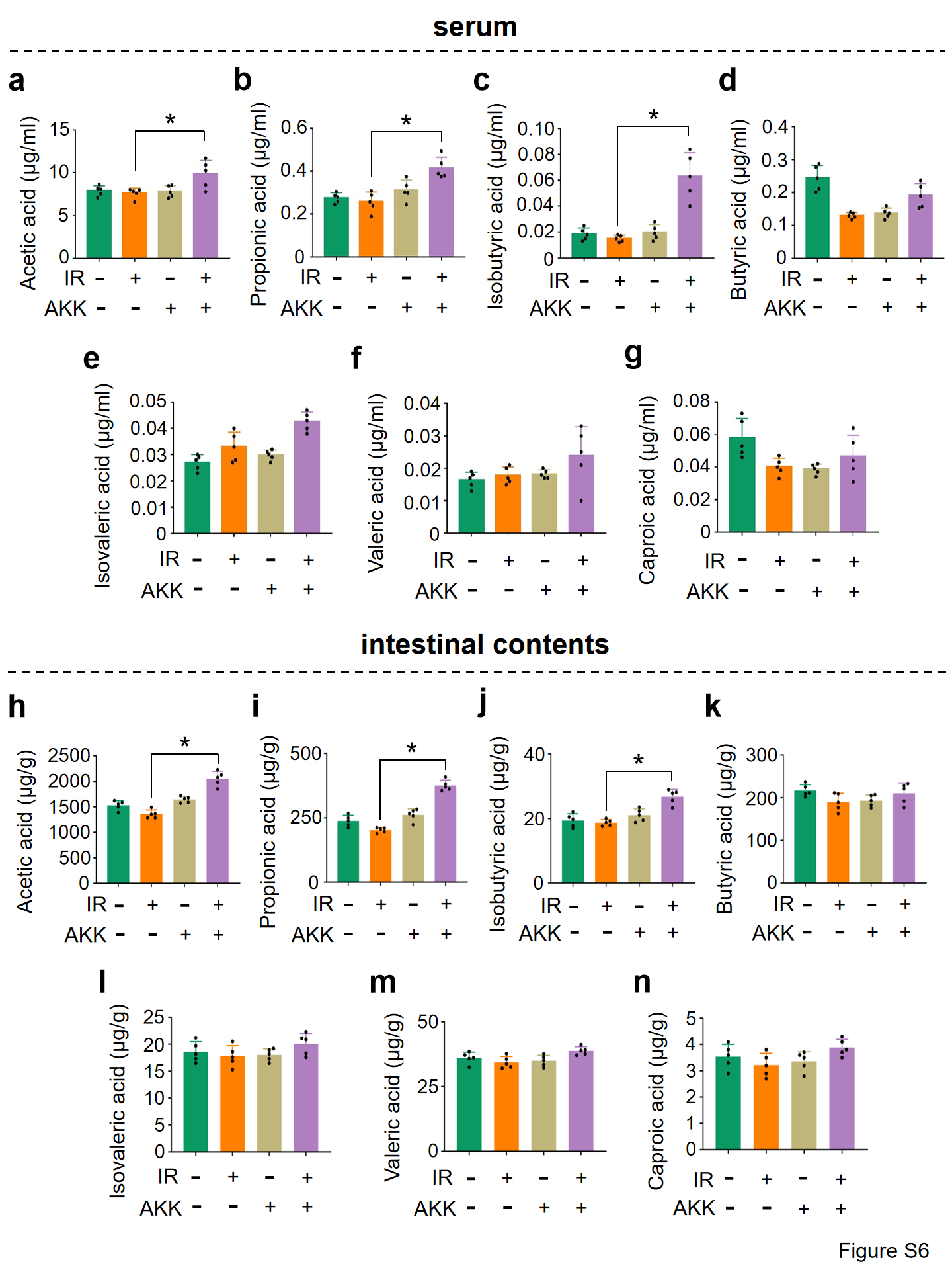

Supplement: Supplemental Material [file KGMI_A_2293312_SM0132.zip › Figure S6 (1).tif]

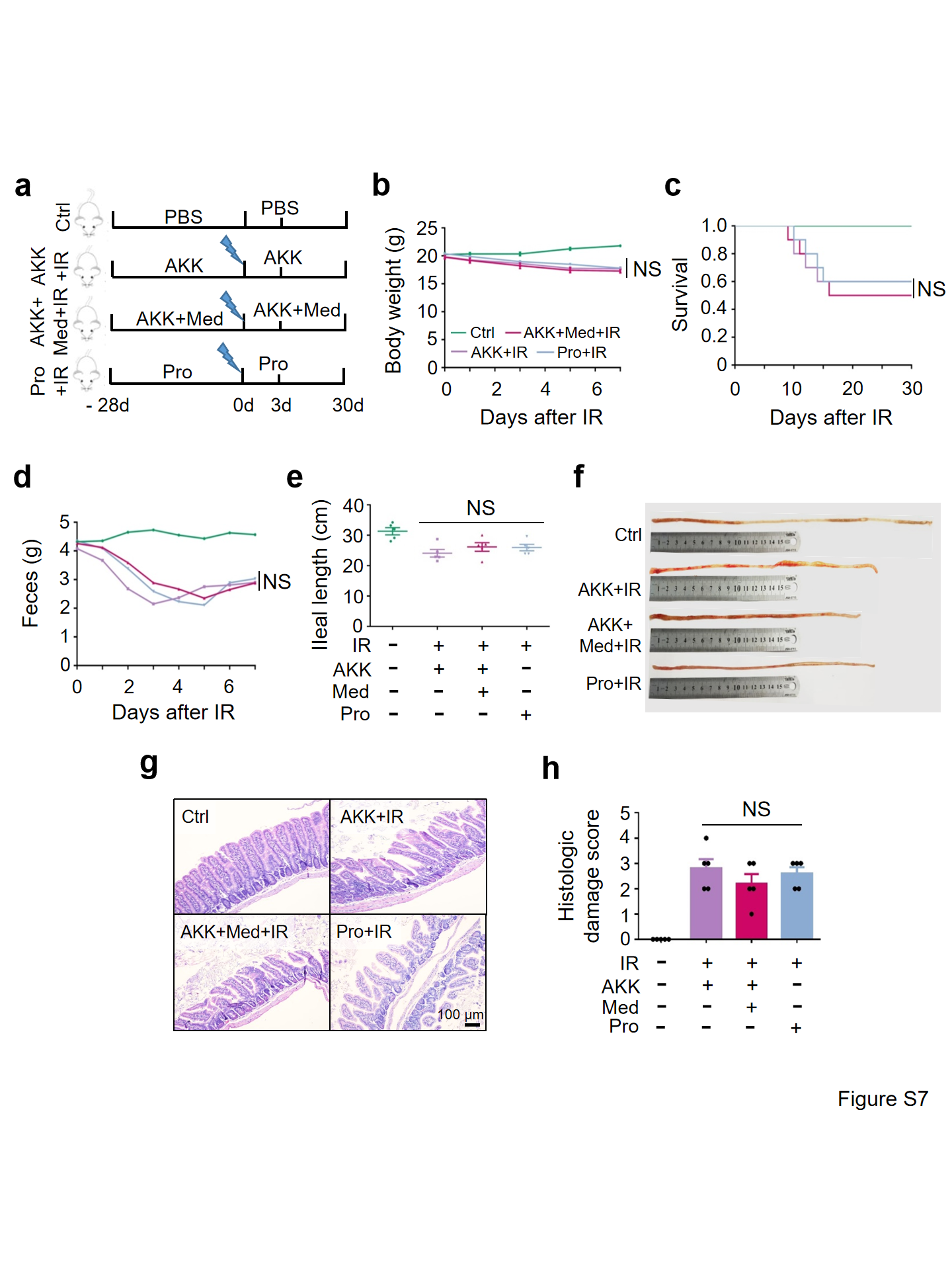

Supplement: Supplemental Material [file KGMI_A_2293312_SM0132.zip › Figure S7 (1).tif]

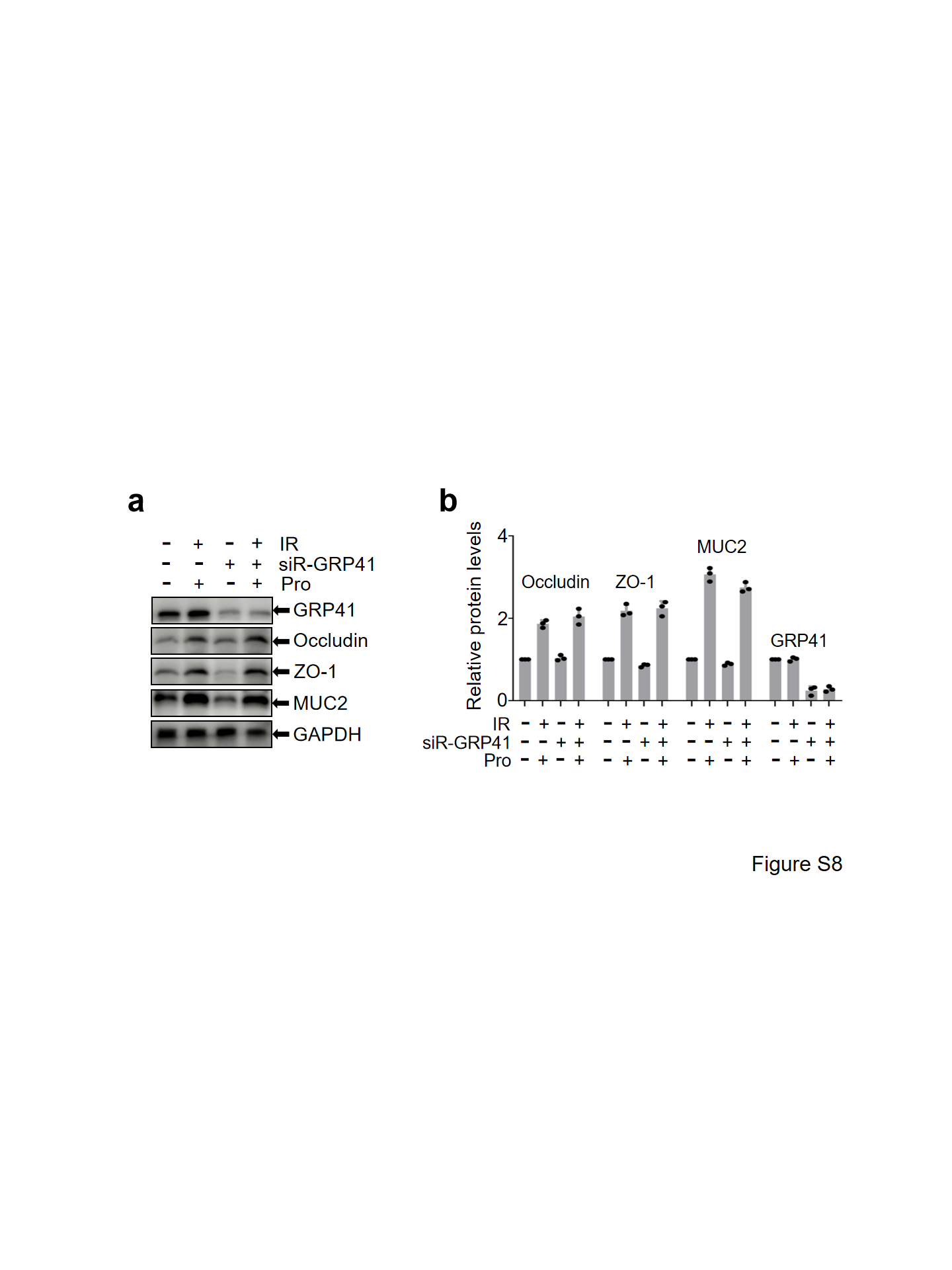

Supplement: Supplemental Material [file KGMI_A_2293312_SM0132.zip › Figure S8.tif]

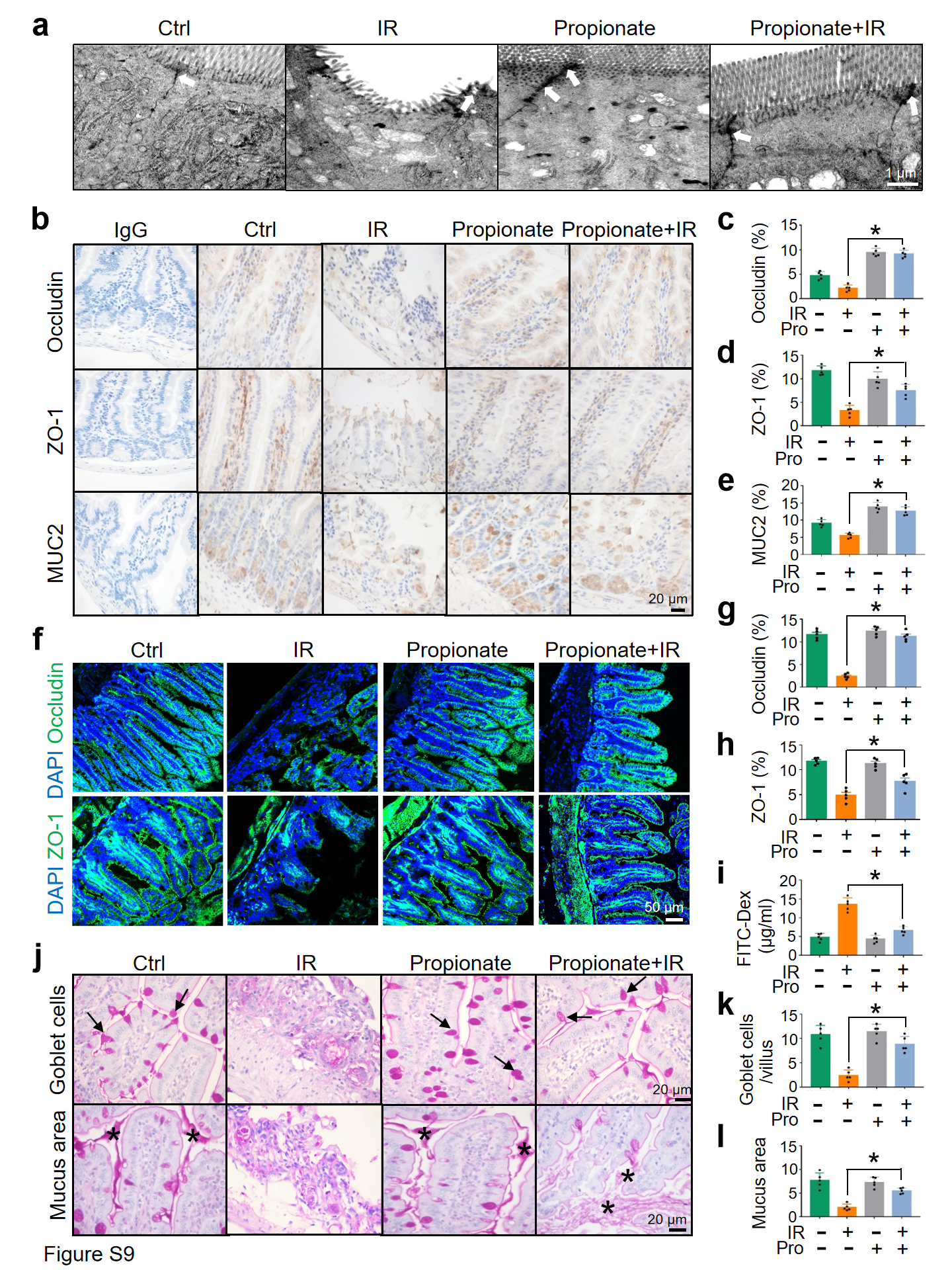

Supplement: Supplemental Material [file KGMI_A_2293312_SM0132.zip › Figure S9.tif]
